# SLEAP: A deep learning system for multi-animal pose tracking

**DOI:** 10.1038/s41592-022-01426-1

**Published:** 2022-04-04

**Authors:** Talmo D. Pereira, Nathaniel Tabris, Arie Matsliah, David M. Turner, Junyu Li, Shruthi Ravindranath, Eleni S. Papadoyannis, Edna Normand, David S. Deutsch, Z. Yan Wang, Grace C. McKenzie-Smith, Catalin C. Mitelut, Marielisa Diez Castro, John D’Uva, Mikhail Kislin, Dan H. Sanes, Sarah D. Kocher, Samuel S.-H. Wang, Annegret L. Falkner, Joshua W. Shaevitz, Mala Murthy

**Affiliations:** 1 Princeton Neuroscience Institute, Princeton University, Princeton, NJ USA; 2 Department of Molecular Biology, Princeton University, Princeton, NJ USA; 3 Department of Ecology and Evolutionary Biology, Princeton University, Princeton, NJ USA; 4 Lewis–Sigler Institute for Integrative Genomics, Princeton University, Princeton, NJ USA; 5 Department of Physics, Princeton University, Princeton, NJ USA; 6 Center for Neural Science, New York University, New York, NY USA; 7 Department of Psychology and Department of Biology, New York University, New York, NY USA; 8 Present Address: The Salk Institute for Biological Studies, La Jolla, CA USA; 9 Present Address: Department of Biomedical Engineering, Johns Hopkins University School of Medicine, Baltimore, MD USA

**Keywords:** Computational neuroscience, Software, Machine learning

## Abstract

The desire to understand how the brain generates and patterns behavior has driven rapid methodological innovation in tools to quantify natural animal behavior. While advances in deep learning and computer vision have enabled markerless pose estimation in individual animals, extending these to multiple animals presents unique challenges for studies of social behaviors or animals in their natural environments. Here we present Social LEAP Estimates Animal Poses (SLEAP), a machine learning system for multi-animal pose tracking. This system enables versatile workflows for data labeling, model training and inference on previously unseen data. SLEAP features an accessible graphical user interface, a standardized data model, a reproducible configuration system, over 30 model architectures, two approaches to part grouping and two approaches to identity tracking. We applied SLEAP to seven datasets across flies, bees, mice and gerbils to systematically evaluate each approach and architecture, and we compare it with other existing approaches. SLEAP achieves greater accuracy and speeds of more than 800 frames per second, with latencies of less than 3.5 ms at full 1,024 × 1,024 image resolution. This makes SLEAP usable for real-time applications, which we demonstrate by controlling the behavior of one animal on the basis of the tracking and detection of social interactions with another animal.

## Abstract (web summary)

SLEAP is a versatile deep learning-based multi-animal pose-tracking tool designed to work on videos of diverse animals, including during social behavior.

## Main

Quantitative measurements of animal motion are foundational to the study of animal behavior^1,2^. Methods for pose estimation, the task of predicting the location of animal body parts in images, have grown in popularity as a state-of-the-art requirement for behavioral quantification across disciplines including neuroscience^3^ and ecology^4^. Although adaptations of deep learning-based approaches originally developed for human pose estimation have made animal pose estimation for single individuals possible^5–7^, reliably tracking multiple, interacting animals and their poses remains a challenging problem, presenting an impediment to studies of social behaviors.

**Fig. 1:**
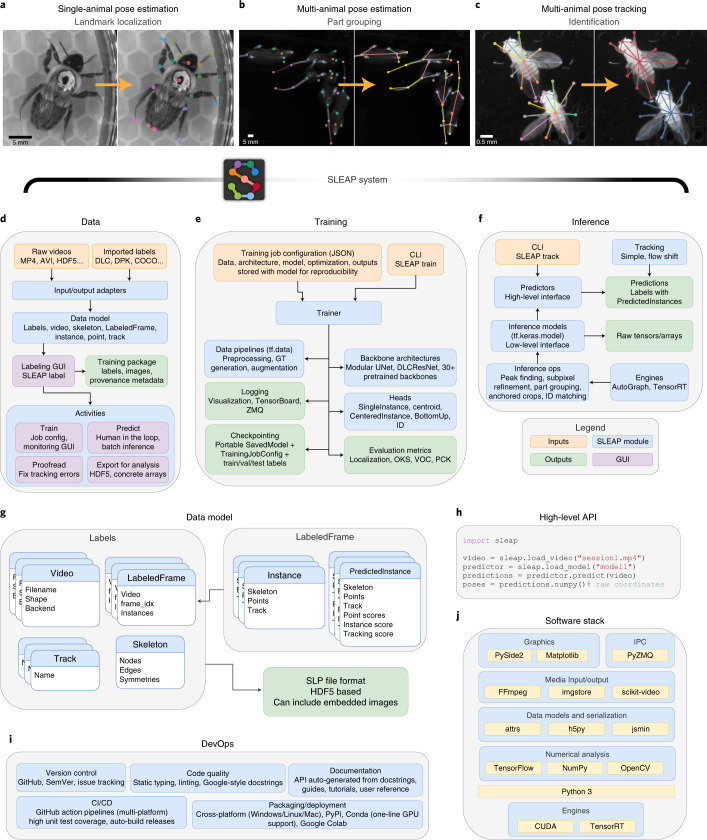
SLEAP is a general-purpose multi-animal pose-tracking system. **a**, Illustration of the part-localization problem. Single-animal pose estimation is equivalent to the landmark-localization task in which there exists a unique coordinate corresponding to each body part. **b**, Illustration of the part-grouping problem. In multi-animal pose estimation, there may be multiple detections of each body part, which must be grouped into sets that correspond to distinct animals. **c**, Illustration of the identity-tracking problem. In multi-animal pose tracking, pose detections must be associated with a unique animal ID that persists across frames. **d**–**f**, Diagram of the submodules in SLEAP, including all major machine learning system components: data annotation, data processing, model configuration (config), model training, model evaluation and inference. DLC, DeepLabCut; DPK, DeepPoseKit; COCO, common objects in context; I/O, input–output; train/val/test, training, validation and test; ops, operations. **g**, Diagram of SLEAP’s data model for describing the structure of both training annotations and predictions in multi-animal pose tracking. **h**, Example of SLEAP’s high-level API for data loading, model configuration, pose prediction and conversion to concrete numeric arrays. **i**, Diagram of development operations (DevOps) practices and components employed in SLEAP’s engineering workflow. CI, continuous integration; CD, continuous deployment. **j**, Diagram of the stack of open-source and modern software libraries that power functionality in SLEAP. IPC, inter-process communication.

Detecting body parts is sufficient for single-animal pose estimation (Fig. 1a), but generalizing to multiple animals requires solutions for assigning detections reliably to individuals both within an image (Fig. 1b) and across frames (Fig. 1c)^3^. While tools have been developed for tracking the identities of multiple animals across consecutive frames^8,9^, a unified approach that simultaneously performs pose estimation and tracking is needed^10^. Existing methods for multi-human pose estimation adopt either a bottom–up (detect parts and then group them into individuals) or top–down (find individuals and then detect parts) strategy, but it is not clear which is better suited for the domain of animals. Tools have been developed that implement one or the other approach^11,12^ for animal pose estimation and tracking, but these methods do not allow the user to compare the two competing approaches.

**Extended Data Fig. 1:**
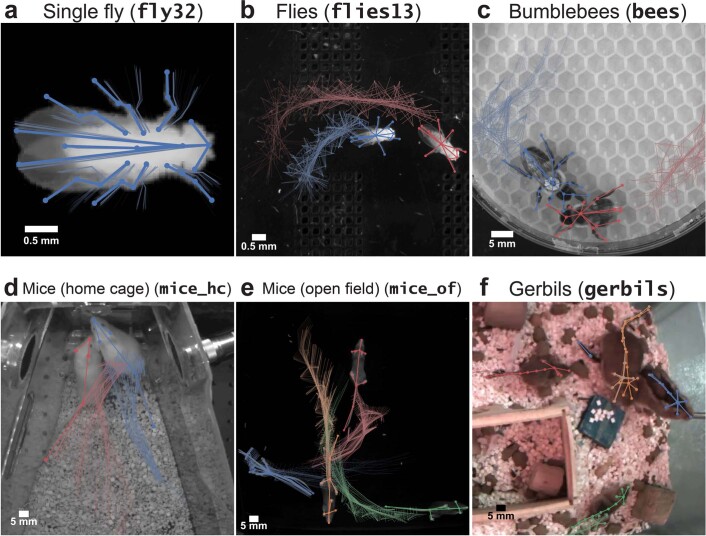
Datasets. **a**, Single fly prealigned. **b**, Flies in 3D printed acoustic recording chamber. **c**, Bees in a behavioral chamber with honeycomb flooring. **d**, Mice in a home cage imaged from above. **e**, Mice in an open field chamber imaged from below. **f**, Gerbils in long-term monitoring home cage.

Here we present Social LEAP (SLEAP), a system for multi-animal pose tracking and the successor of the single-animal pose-estimation method LEAP^6^. SLEAP is a general-purpose framework developed from the ground up and meets the needs of the entire multi-animal pose-tracking workflow, including interactive labeling, training, inference and proofreading. SLEAP implements both top–down and bottom–up approaches, animal identity tracking through motion or appearance models and over 30 state-of-the-art neural network backbones and modular network architectures. We demonstrate the importance of this flexibility by evaluating SLEAP across seven datasets with different species, numbers of animals, body parts, imaging conditions and environments (Extended Data Fig. 1 and Supplementary Video 1). We show that SLEAP is accurate (<0.11 mm for flies, <3.3 mm for mice in 90% of the data), data efficient (<200 labels for 90% peak accuracy in flies and mice), fast to train (90% peak accuracy within 4.4 min without pretraining) and fast to predict (up to 804 frames per second (FPS) without downsampling). SLEAP is able to perform end-to-end tracking of high-resolution multi-animal data at low latencies (<3.5 ms for 1,024 × 1,024-pixel images), making it compatible with real-time processing. To demonstrate how SLEAP can enable experimental paradigms intractable without reliable real-time multi-animal tracking, we implement a closed-loop system to optogenetically control the behavior of one animal on the basis of social interactions with another animal detected in real time. Finally, we have made SLEAP available at https://sleap.ai, together with an accessible user interface, open-source code, extensive documentation and tutorials as well as all datasets and annotations to establish a reproducible and comprehensive multi-animal pose-tracking benchmark.

## Results

### SLEAP is a complete framework for multi-animal pose tracking

**Extended Data Fig. 2:**
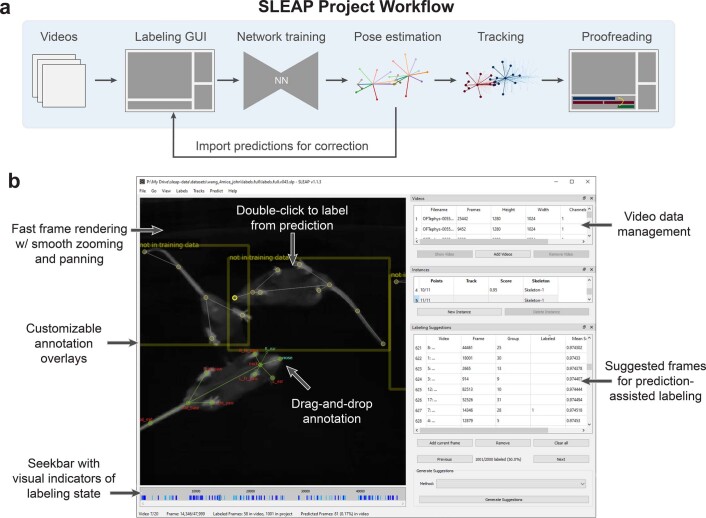
SLEAP labeling workflow. **a**, Schematic of the SLEAP labeling workflow, from raw data to tracked videos. **b**, Screenshot of interactive SLEAP labeling interface. This interface can also be used for inspection and proofreading.

The SLEAP multi-animal pose-tracking system is composed of submodules that can be configured to enable a workflow starting from data input and resulting in trained pose-estimation models and pose-tracked videos (Extended Data Fig. 2a). Such a system is needed to make SLEAP general purpose or, in other words, to enable it to flexibly perform well on any dataset or training regime. SLEAP implements an input–output layer that supports data input in raw video or array format as well as importing annotations from other pose-tracking software^5,7^ and standardized formats^13^ (Fig. 1d). Once imported, data can be labeled interactively using a versatile graphical user interface (GUI) (Extended Data Fig. 2b and Supplementary Video 2) that can then export images and annotations as a single-file ‘training package’ to facilitate remote training and data sharing. Predictions from trained models can be imported, sorted based on prediction score and used to initialize ground truth labels for frames that performed poorly, requiring less time to correct than de novo labeling. This functionality enables a human-in-the-loop workflow in which the user alternates between labeling and training models to produce progressively more accurate predictions. The GUI also provides functionality for launching and monitoring training, proofreading predicted data and exporting raw positional data in a format convenient for analysis.

**Extended Data Fig. 3:**
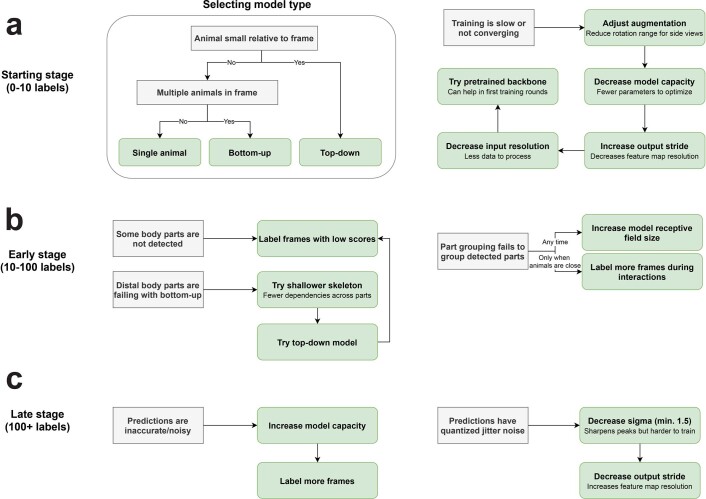
Troubleshooting workflows. **a**, Schematics of starting stage workflows. Before the first training round, it is important to select the appropriate model type and adjust basic training parameters as needed. **b**, Schematics of early stage workflows. Poor performance is expected with few labeled frames, but certain types of errors may be mitigated by adjusting basic model parameters, such as receptive field size. **c**, Schematics of late stage workflows. Fine tuning performance once enough frames are labeled may be accomplished by trading off speed for accuracy, such as by increasing the resolution of the model features.

SLEAP supports multiple approaches to solving pose-estimation problems as well as more than 30 neural network architectures to learn from data (Fig. 1e). To enable this flexibility as well as to ensure reproducibility, we implemented a configuration system that captures all hyperparameters related to model creation and training. These configuration files describe data-preprocessing steps, neural network architecture, optimization settings and output formats. This can be used to reproduce training results and is used to document the inputs and outputs of a saved model. We provide several built-in configuration profiles that are applicable for a wide range of use cases and datasets as well as online documentation and troubleshooting workflows for common problems that users may encounter (Extended Data Fig. 3).

Once trained, SLEAP models can be used to predict poses from previously unseen data (Fig. 1f). We implemented efficient approach-specific algorithms in graphics processing unit (GPU)-accelerated code, which is automatically added based on the model configuration. This enables high-performance inference either through a command-line interface (CLI) or through low-level or high-level application programming interfaces (APIs) to enable custom applications that use SLEAP as a component. Inference modules provide low-level functionality in native TensorFlow code for GPU compatibility, including peak finding, subpixel refinement and other operations necessary for complex multi-stage models. Tracking modules can optionally be enabled to associate poses over time (Fig. 1c) or disabled when labeling discontiguous frames. Results from any mode of inference can be saved with associated metadata, allowing the user to open predictions in the GUI for convenient inspection and proofreading.

As part of the framework, we developed a standardized data model that encompasses the needs of general-purpose multi-animal pose tracking (Fig. 1g and Supplementary Table 3). This data model describes all structures used in labeling, training and inference, including properties that are specific to the multi-animal setting (for example, Track). Importantly, this data model is format agnostic, which enables standardization and sharing of animal pose data regardless of provenance. We implement this data model within SLEAP and develop a self-contained format in which it can be saved to a single, portable file that can optionally include image data.

SLEAP can be used entirely through its GUI with no programming required; however, we also expose convenient high-level APIs (Fig. 1h) that can be used to build applications and extensions that use SLEAP^14–16^. To support the engineering complexities of a large-scale software system, we adopted industry-standard practices for software engineering and developer operations (Fig. 1i). We use automated tools for versioning, continuous integration, packaging, distribution and documentation to enable a reliable, reproducible and documented software package. This allows SLEAP to work across platforms and maximizes the validity and future reproducibility of scientific results derived from its use.

SLEAP is open-source software that builds upon a large number of other state-of-the-art software packages for numerical analysis and deep learning (Fig. 1j). Implemented entirely in Python, SLEAP takes advantage of current and future developments in each layer of its infrastructure.

### Fast, efficient and accurate animal pose estimation

We use the mean average precision (mAP) metric from the human pose-estimation literature to summarize performance while taking into account animal size, visibility of body parts and uncertainty in human-labeling precision^17^. We implement this calculation with the assumption that all animal landmarks are as ‘easy’ to label as the most unambiguous human landmark (the eye). This provides a lower bound on the true accuracy of these models.

**Fig. 2:**
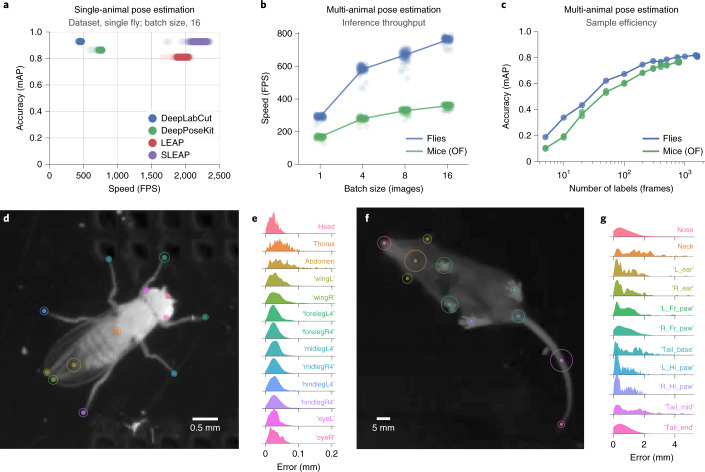
SLEAP is fast, efficient and accurate. **a**, Speed versus accuracy of different animal pose-estimation frameworks on a single-animal dataset^6^. Points correspond to sampled measurements of batch-processing speed over 1,280 images with the highest-accuracy model replicate from each framework. Accuracy was evaluated on a held-out test set of 150 images. **b**, Speed versus batch size for multi-animal datasets. Points correspond to sampled measurements of batch-processing speed over 1,280 images and five replicates. OF, open field. **c**, Sample efficiency across multi-animal datasets. Points indicate accuracy of model training replicates on the held-out test set. **d**–**g**, Body part-wise landmark-localization accuracy. Circles denote the 95th percentile of localization errors, and histograms correspond to full error distribution evaluated on held-out test sets (*n* = 150 frames for flies, *n* = 100 frames for mice). L, left; R, right; hi, hind; fr, front. Source data

The single-animal pose-estimation problem (Fig. 1a) that previous frameworks were developed to solve is a core component of SLEAP. To evaluate how our system performs relative to previous approaches, we applied SLEAP to a published single-animal dataset^6^. When compared to DeepLabCut^5^, DeepPoseKit^7^ and LEAP^6^, we found that SLEAP achieves comparable or improved accuracy (mAP scores of 0.927 versus 0.928 for SLEAP and DeepLabCut, respectively) at prediction speeds that are several times faster (2,194 versus 458 FPS) (Fig. 2a).

Next, we evaluated how SLEAP performs on multi-animal datasets of both flies and mice. We found that SLEAP is able to reach peak inference speeds of 762 and 358 FPS for flies and mice, respectively (Fig. 2b), while achieving 50% peak accuracy with as few as 20 labeled frames and 90% accuracy with 200 labeled frames (Fig. 2c), comparable to the efficiency of previous frameworks on single-animal data^6^. Inspecting the distributions of landmark-localization errors, we found that SLEAP is able to identify the ground truth location of body parts of both flies (Fig. 2d,e) and mice (Fig. 2f,g) with high accuracy at anatomical scales, with 95% of estimates within 0.084 mm (3.2% of body size) for flies and 3.04 mm (3.7% of body size) for mice. SLEAP recovers poses at high mAP scores (0.821 for flies and 0.774 for mice) as compared to top scores previously reported on multi-person pose-estimation benchmarks (0.774 (ref. ^18^)).

### Flexible approaches to multi-instance pose estimation

SLEAP implements two classes of approaches for solving the multi-instance pose-estimation problem (Fig. 1b): the bottom–up and the top–down approach (Supplementary Video 3). These approaches differ in how they model the relationship between animal instances and their body parts and come with different performance trade-offs. In particular, we note that SLEAP is agnostic to the specific neural network architecture underlying a particular model and can use any fully convolutional architecture with either approach. Note that the nomenclature of bottom–up and top–down refers to the conceptual organization of the algorithmic approaches, and does not refer to camera orientation (either can be used with any camera placement).

**Fig. 3:**
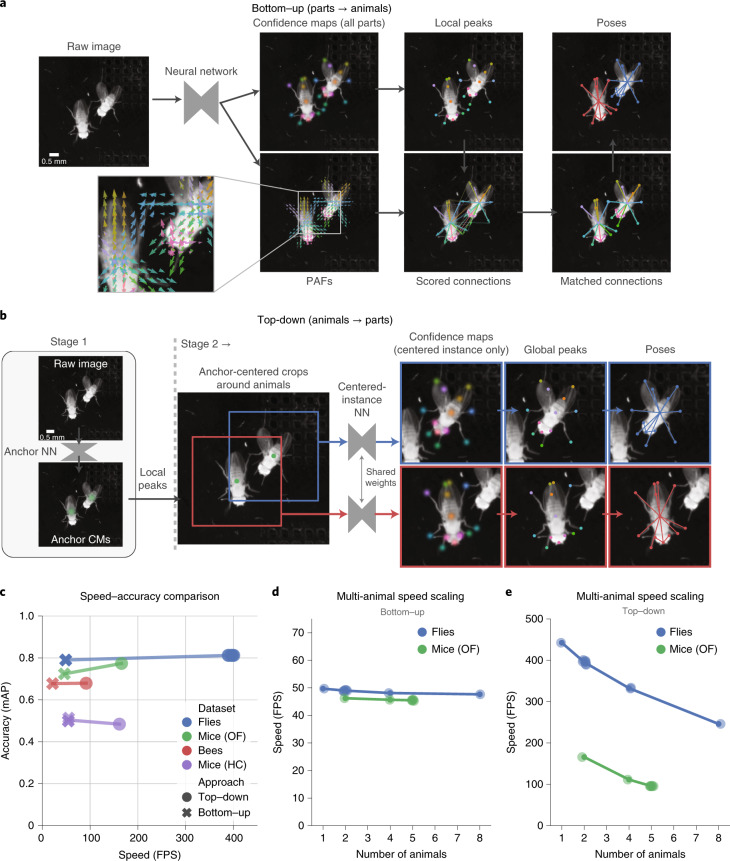
Multi-animal pose-estimation approaches in SLEAP. **a**, Workflow for the bottom–up approach. From left to right: a neural network takes an uncropped image as input and outputs confidence maps and PAFs; these are then used to detect body parts as local peaks in the confidence maps and score all potential connections between them; on the basis of connection scores, a matching algorithm then assigns the connections to distinct animal instances. **b**, Workflow for the top–down approach. From left to right: the first-stage neural network (NN) takes an uncropped image as input and outputs confidence maps for an anchor point on each animal; the anchors are detected as local peaks in the confidence maps (CMs); a centered crop is performed around each anchor point and provided as parallel inputs to the second-stage neural network; the network outputs confidence maps for all body parts only for the centered instance, which are then detected as global peaks. **c**, Speed versus accuracy of models trained using the two approaches across datasets. Points denote individual model replicates and accuracy evaluated on held-out test sets. Top–down models were evaluated here without TensorRT optimization for direct comparison to the bottom–up models. HC, home cage. **d**, Inference speed scaling with the number of animals in the frame for bottom–up models. Points correspond to sampled measurements of batch-processing speed (batch size of 16) over 1,280 images with the highest-accuracy model for each dataset. **e**, Inference speed scaling with the number of animals in the frame for top–down models. Points correspond to sampled measurements of batch-processing speed (batch size of 16) over 1,280 images with the highest-accuracy model for each dataset. Top–down models were evaluated here without TensorRT optimization for direct comparison to the bottom–up models. Source data

In the bottom–up approach, all body parts are detected within an image and then grouped into animals based on their connectivity (Fig. 3a). This approach has the advantage that it only requires a single pass through the neural network, which outputs multi-part confidence maps and part affinity fields (PAFs)^19^, a set of vector fields that represent spatial relationships between pairs of body parts. The multi-part confidence maps are used to recover individual body part coordinates, which are then grouped into complete animals by evaluating the connectivity score between pairs of detected points for each body part type. We implement this approach with GPU-accelerated operations for evaluating and matching potential connections efficiently. This approach explicitly models animal morphology by describing its skeleton as a directed tree (Supplementary Note).

In the top–down approach, we first detect all animals and then locate their body parts (Fig. 3b). In the first stage, a neural network detects an anchor point (for example, centroids) on each animal, which can then be recovered via local peak finding. In the second stage, the anchor points are used to generate anchor-centered sub-images cropped around each animal, which are then provided as input to a second neural network. This centered-instance network is trained to predict unimodal confidence maps only for the centered animal even if other animals are visible in the sub-image. The set of sub-images in each frame are processed in parallel, and part coordinates are estimated from confidence maps through global peak finding. In contrast to the bottom–up approach, this approach models animals implicitly through the use of an animal-centered sub-image, which encodes a spatial prior on the relative positioning of body parts in the sub-images.

To evaluate the performance of each approach, we trained models on four different multi-animal datasets with flies, bees, mice in a high-contrast featureless arena (open field) and mice in their cages with bedding and other visual features (home cage). For all datasets except the one with mice in their home cage, we observed higher accuracy with top–down models and considerably higher speed across all datasets (Fig. 3c). While top–down models are typically faster than bottom–up models with few animals, their performance varies with the number of animals. Bottom–up models scale efficiently with increasing numbers of animals owing to their single-stage construction (Fig. 3d), while top–down models (Fig. 3e) scale linearly as their second stage will run once per animal. These results demonstrate that the bottom–up approach may be advantageous in datasets in which there are many animals that occupy a large fraction of the field of view, while the top–down approach is preferable for datasets with few animals.

### Flexible configuration of neural network architectures

**Fig. 4:**
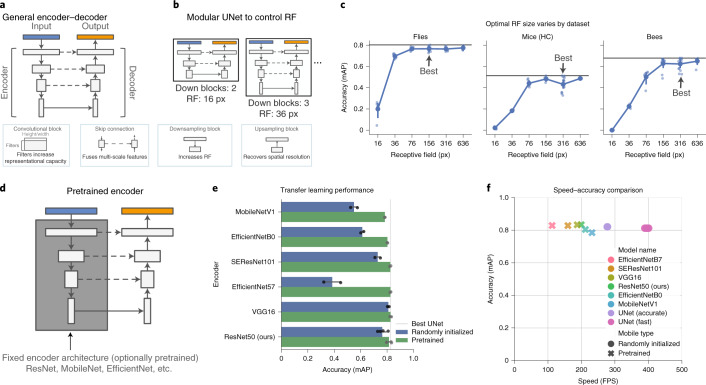
Neural network architectures are highly configurable in SLEAP. **a**, Schematic of the general encoder–decoder neural network architecture, which is composed of standard blocks with different properties (bottom). **b**, Schematic of the modular version of UNet in SLEAP, which can be configured to control the maximum RF of the network by varying the number of downsampling blocks at the cost of more computations. px, pixels. **c**, Accuracy of UNets configured at different RFs across datasets. Points correspond to model training replicates, and the black line denotes the maximum accuracy achieved across all replicates (*n* = 3–5 per RF size per dataset, total of *n* = 115 models). Accuracy was evaluated on held-out test sets. **d**, Schematic of how SLEAP can use fixed network architectures as the encoder backbone to enable transfer learning. **e**, Accuracy of encoders with commonly used network architectures initialized with random or pretrained weights (transfer learning). Bars and error whiskers (mean and 95% confidence interval) correspond to top–down model training replicates (*n* = 3–5 per model architecture) on a held-out test set of the fly dataset. The gray line denotes the randomly initialized modular UNet baseline. MobileNetV1, MobileNet version 1. **f**, Speed versus accuracy comparison of the pretrained encoder and UNet model variants. Points correspond to average speed evaluated over 1,280 images for the most accurate model of each category. Accuracy was evaluated on the held-out test set of the fly dataset. Source data

Optimal neural network architecture design is an area of active research, and little is known about optimal architectures in applied domains such as animal pose estimation. SLEAP can be configured to use any fully convolutional neural network architecture backbone while being agnostic to the specific approach employed (for example, top–down, bottom–up), making it an ideal platform for studying the performance of neural network architecture properties. To this end, we implemented a generic formulation of the encoder–decoder architectural pattern (Fig. 4a). These types of models are composed of building blocks that imbue the network with different capabilities, such as increasing the maximum receptive field size (RF) to enable the model to learn and reason about image features across spatial scales^20^.

**Extended Data Fig. 4:**
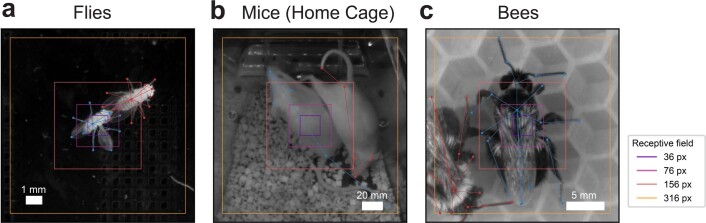
Receptive field sizes. **a**, Receptive field sizes overlaid on example frame from flies dataset. **b**, Receptive field sizes overlaid on example frame from mice dataset. **c**, Receptive field sizes overlaid on example frame from bees dataset. Source data

The primary architecture type used in SLEAP is a modular version of UNet, a simple encoder–decoder architecture commonly used in biomedical applications^21^. UNet can be configured with a variable number of downsampling blocks to modulate the RF size of the network, controlling its ability to reason over larger regions of the image at the cost of increased memory and computation time (Fig. 4b). In general, larger receptive fields require larger networks and thus are slower to train and evaluate. To evaluate the effect of modulating the RF size of neural network architectures, we evaluated the pose-estimation accuracy of different model configurations across datasets (Extended Data Fig. 4). We found that increasing RF size improved accuracy, but the relative improvement saturated at different points depending on the dataset (Fig. 4c). These results indicate that these networks can be configured with the RF size best suited to capturing the image features unique to the dataset, thereby achieving high accuracy while reducing computational and memory costs of larger models. We note that the scale of these image features is determined by a combination of animal size, imaging resolution and the target morphological features.

**Extended Data Fig. 5:**
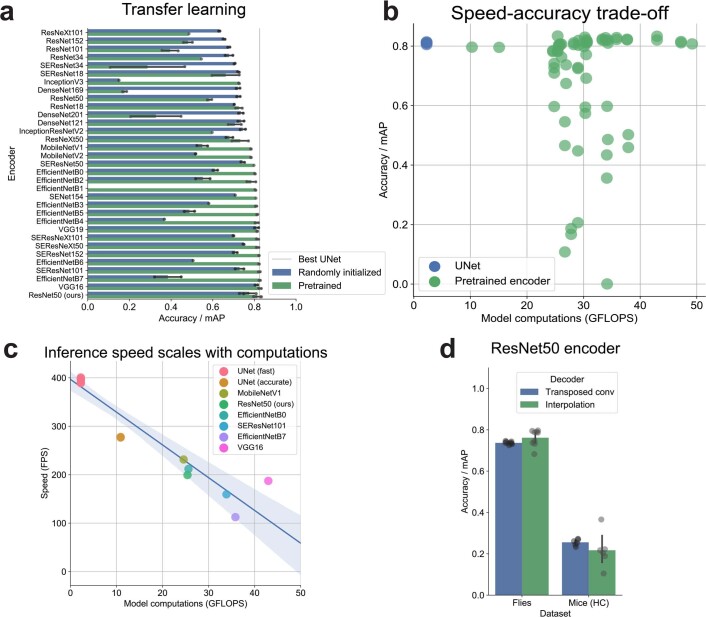
Pretrained encoder backbone models. **a**, Transfer learning performance across all tested pretrained encoder model architectures. Accuracy evaluated on held-out test set of flies dataset using the top-down approach (n = 2-5 models per architecture and condition; 125 total models). **b**, Speed versus accuracy trade-off across all tested pretrained encoder model architectures as compared to optimal UNet. Accuracy evaluated on held-out test set of flies dataset using the top-down approach. Model floating point operations (GFLOPS) derived directly from configured architectures (n = 2-5 models per architecture and condition; 68 total models). **c**, Relationship between inference speed and computations. Points correspond to speed of the best model replicate for each architecture. Line and shaded area denotes linear fit and 95% confidence interval. **d**, Accuracy of our implementation of DLC ResNet50 with different decoder architectures. Points denote model training replicates (n = 3-5 models per condition; 30 total models). Source data

**Extended Data Fig. 6:**
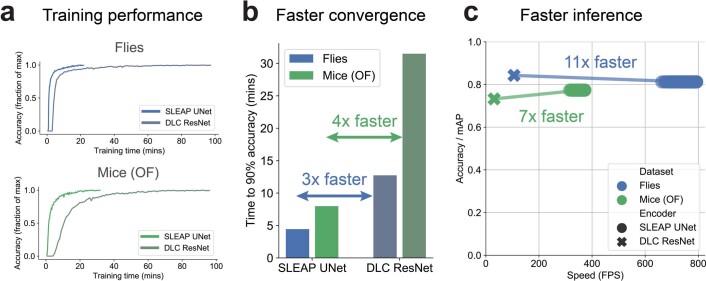
SLEAP UNet versus DeepLabCut ResNet performance for multi-animal pose estimation. **a**, Relative accuracy as a function of training time for flies and mice (OF) datasets. Accuracy evaluated on a held-out test set by using model checkpoints saved at every epoch (checkpointing time not included). Accuracy is normalized to the maximum accuracy (mAP) achieved over all epochs. **b**, Summary of training efficiency across different model types and datasets. Time is the minimum training time from (a) required to reach 90% peak accuracy. **c**, Speed versus accuracy trade-off of using SLEAP UNet versus DLC ResNet models for multi-instance pose estimation. Points denote benchmark replicates and lines connect means per condition. DLC ResNet in all panels refers to an implementation of a ResNet50-based architecture configured to mimic the default configuration in DeepLabCut.

Other popular tools for animal pose estimation, however, make use of standard neural network architectures for which pretrained weights are available to enable transfer learning, which is thought to improve performance for datasets that have few labels^5,22^. SLEAP allows for the configuration of these types of models by using them as the backbone for the encoder portion of the model and connecting intermediate-layer activations with the decoder to recover spatial resolution (Fig. 4d). To test the effectiveness of transfer learning, we evaluated the performance of a large number of state-of-the-art network architectures with either randomly initialized or pretrained initial weights (Extended Data Fig. 5). We found that transfer learning typically results in accuracy improvements over random initialization but does not confer advantages over the optimal randomly initialized UNet (Fig. 4e and Extended Data Fig. 5a). While pretrained encoder models can achieve high accuracy, we find that these general-purpose architectures come at the cost of a considerable increase in computations (Extended Data Fig. 5b). This results in slower inference speeds (Extended Data Fig. 5c) at the same accuracy as simpler and more lightweight models that do not require pretraining (Fig. 4f). We find that these results are reflected in both 3–4× longer training times and 7–11× slower inference speeds in architectures such as those used in DeepLabCut (Extended Data Fig. 6).

### Tracking identities via temporal and appearance models

To address the identification problem (Fig. 1c), SLEAP implements two classes of techniques for maintaining animal identities across frames using either temporal-based or appearance-based cues (Supplementary Video 4).

**Fig. 5:**
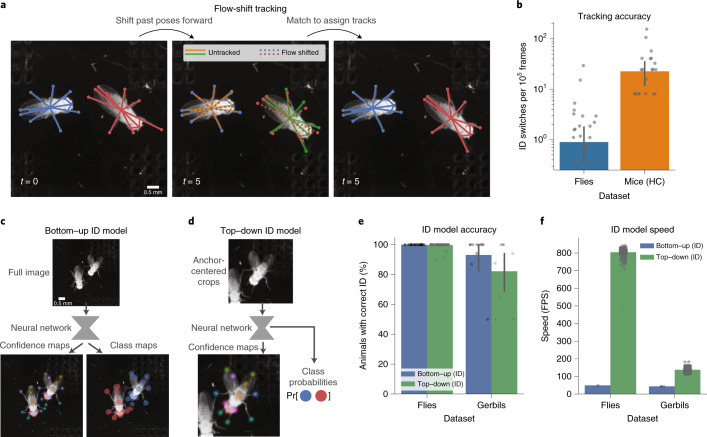
Tracking and identification using temporal and appearance models in SLEAP. **a**, Schematic of flow-shift tracking in which SLEAP associates poses across frames by using temporal context to predict where past poses will be in the current frame, allowing identities to be matched across time. **b**, ID-switching accuracy of flow-shift tracking over entire proofread datasets. Points correspond to ID-switching rate per 100,000 frames for individual videos in each dataset (*n* = 11.7 million frames over 87 videos for flies; *n* = 367,000 frames over 30 videos for mice). Bars and error whiskers correspond to mean and 95% confidence intervals. **c**, Schematic of the bottom–up ID approach, in which each distinct animal ID is treated as a class that is characterized by distinctive appearance features. **d**, Schematic of the top–down ID approach (only the second stage is shown), in which crops are used to predict confidence maps for the centered instance as well classification probabilities for matching instances to IDs (probability vector denotes with Pr[] in schematic). **e**, ID model accuracy across approaches and datasets. Points correspond to the fraction of animals identified correctly in each video in the held-out test sets (*n* = 150 frames for flies, *n* = 42 frames for gerbils). Bars and error whiskers correspond to the mean and 95% confidence intervals. **f**, Inference speed of each approach across datasets. Points correspond to sampled measurements of batch-processing speed over 1,280 images with the highest-accuracy model for each approach and dataset. The fastest batch size for each approach was selected (32, bottom–up; 16, top–down). Source data

First, we implemented a flow-shift-based tracking approach^23^ that uses optical flow to estimate the displacement of poses across frames. Past poses shifted onto the current frame can then be used to associate previous pose detections with new ones (Fig. 5a). We note that our implementation uses a simple optical flow algorithm that does not require model training, enabling users to perform tracking with no additional labeling of consecutive frames. Using SLEAP’s proofreading GUI to identify and correct identity switches in two large multi-session datasets, we find that identification (ID) switches are rare across datasets (0.91 and 22.7 switches per 100,000 frames for flies and mice, respectively) (Fig. 5b). We corrected ID switches in only 62 of 11.7 million frames for flies and 83 of 367,000 frames for mice, which took only minutes to identify and proofread with our GUI.

The inherent drawback of temporal tracking, however, is that errors such as identity switches propagate over time. This results in incorrect identity assignments for long spans of time even in cases in which tracking errors occur rarely, making this technique less useful for very long videos (which would be intractable to proofread) or real-time applications (which cannot be proofread). To address this, we developed extensions to our multi-instance pose-estimation models that leverage appearance as a cue for identity assignment on a single-frame basis.

In our bottom–up ID models, we replace the PAFs of the standard approach with multi-class segmentation maps, a representation similar to those of conventional segmentation tasks (Fig. 5c). These models predict the probability that each unique animal class is occupying the region surrounding each landmark, enabling identity assignment through an optimal assignment of the probabilities at each detected landmark location. The class maps make PAFs unnecessary as grouping is implicit in the ID assignment.

In our top–down ID models, we employ a technique similar to that in previously described appearance models^8^. Here, we predict animal class probabilities for each animal-centered sub-image in addition to pose (Fig. 5d). In these models, classification probabilities are computed from the features output by the deepest layer in the network. These classification probabilities are then used as scores for an optimal matching of instances to unique animal IDs. Bottom–up and top–down ID models both produced high accuracy for flies (99.7%, bottom–up; 100%, top–down; Fig. 5e).

To further test the ability of these approaches to correctly identify individuals in more difficult situations, we used multi-day movies of four gerbils in a home cage. This dataset presents challenges including lens distortion, suboptimal focus, motion blur and highly variable illumination. The experimental conditions are challenging as gerbils frequently engage in huddling, resulting in heavy occlusion, the home cage bedding visually blends with the animals’ fur and enrichment objects occlude the animals from the camera. Unlike the other datasets that we used, which consist of sessions on the order of tens of minutes, this dataset was recorded continuously over a period of days so that even rare identity switching would make proofreading laborious as errors would be difficult to identify and correct, in addition to propagating over millions of frames. This is further compounded by having four animals, which considerably increases the number of possible incorrect combinations of identity assignments. This dataset is ideally suited for appearance-based ID models that can leverage variability in body morphology and fur patterning across animals as distinguishing features and do not rely on temporal dependencies across frames, thereby guaranteeing that ID errors will not be propagated over time, which effectively eliminates the need for proofreading. We find that these models perform well with this dataset, despite the challenging conditions (82.2%, bottom–up; 93.1%, top–down; Fig. 5e).

Next, we measured the speed of the ID models to evaluate their batch inference performance. We found that, while our bottom–up ID models exhibited performance similar to that of their counterparts without the ID branch (49 and 43 FPS for flies and gerbils, respectively), the top–down ID models exhibited the highest performance of all models that we tested, reaching up to 137 and 804 FPS for gerbils and flies, respectively (Fig. 5f). As these models are end to end, these performance measures correspond to the entire inference pipeline, demonstrating that SLEAP is capable of tracking 13 landmarks on two animals and assigning unique identities from raw high-resolution (1,024 × 1,024) frames at over 800 FPS with no downsampling or any other preprocessing or postprocessing required.

While the appearance-based approach has the advantage of not propagating identity errors (Supplementary Video 4), it comes with the trade-off that it requires animals with sufficiently distinctive appearance cues such that they can be manually identified during labeling. By contrast, temporal models do not require additional labeling or training, can be used with visually hard-to-distinguish animals and can work downstream of any standard pose-estimation approach. We offer both approaches in SLEAP.

### Detection and control of behavior in real time

Real-time applications that use feedback on animal pose require a low-latency solution for image capture, pose estimation and feedback output. To measure the time required to estimate animal pose in a single image, we measured the single-frame (batch size of 1) inference latency of these models and found that they were able to produce predictions with delays as short as 3.2 ms (312 FPS, Fig. 5f). As compared to previous approaches for real-time single-animal pose estimation, which achieve a latency of 14 ms with smaller images on similar hardware^24^, SLEAP can achieve a latency of 3.2 ms with full-resolution images (1,024 × 1,024 pixels), 13 body parts and multi-animal tracking.

**Fig. 6:**
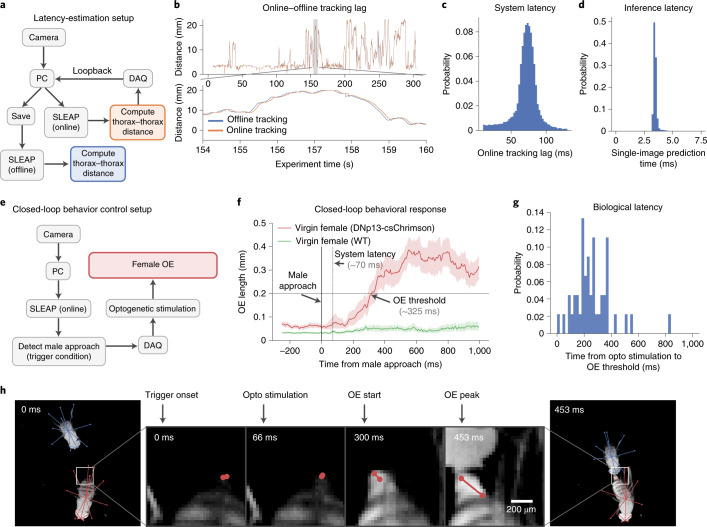
SLEAP can detect social behavior for real-time control. **a**, Schematic of hardware setup for detecting poses, calculating thorax–thorax distance and estimating round-trip latency through a DAQ loopback. PC, personal computer. **b**, Lag between online and offline distance traces estimates round-trip system latency. **c**, Distribution of round-trip system latency estimated by aligning 1-s segments between offline and online traces (mean = 71.0 ms, s.d. = 17.0 ms, *n* = 50,000 1-s segments). **d**, Distribution of end-to-end top–down ID model inference latency for single images (mean = 3.45 ms, s.d. = 0.16 ms, *n* = 1,280 images over five replicates). **e**, Hardware setup for detecting poses, trigger condition (male approach), optogenetic stimulation (of DNp13) and control of virgin female behavior (OE). **f**, Female behavioral response (change in OE length) to male approach-triggered optogenetic activation of DNp13 neurons expressing csChrimson (red) or in virgin WT females (green). The line and shaded regions denote mean and 95% confidence intervals (*n* = 48 bouts, DNp13; *n* = 282 bouts, WT). **g**, Distribution of latency from optogenetic (opto) stimulation onset to OE threshold, indicating the biological latency of the system (mean = 249.0 ms, s.d. = 148.1 ms, *n* = 48 bouts). **h**, Example closed-loop behavioral control event. From left to right: male in approach pose at condition trigger onset; optogenetic stimulation onset; start of female OE response to optogenetic stimulation; peak of female OE response with male still in close proximity. Source data

We next developed a hardware setup to measure the full end-to-end latency for real-time feedback on animal pose. First, we evaluated the latency of the entire closed-loop system using online SLEAP tracking (Fig. 6a). In this setup, a high-precision data acquisition (DAQ) triggers camera frame capture and records the exposure time for synchronization. Frames are sent to both a video encoder for offline processing and to an online SLEAP pose predictor running in parallel. We use the online predictions to encode a pose-derived social feature (thorax to thorax distance) in an analog output signal that is sent through the DAQ and read back in through a loopback connection (Fig. 6b). The delay between the computation of social pose features offline versus online can be used to estimate the full-system latency, which includes overhead from hardware communication and other software layers. We estimate that our system exhibits a 70-ms latency from the time when the frame is captured to when an output signal can be generated based on predicted poses (Fig. 6c), and only about 3 ms (Fig. 6d) are taken up by SLEAP model inference, suggesting that more optimized hardware and software could achieve lower latencies.

Next, we modified the setup to test online SLEAP pose tracking to control the behavior of one animal based on the behavior of its socially interacting partner (Fig. 6e). To do this, we expressed a light-activated cation channel (CsChrimson) in a subset of fly neurons (called DNp13 (ref. ^25^) or pMN1) that controls ovipositor extrusion (OE) in females, a rejection behavior normally produced by mated females during courtship^25,26^. We selected this behavior as it is rarely elicited by unmated, virgin female flies during courtship. To drive OE behavior in virgin females, we activated DNp13 neurons using optogenetics contingent on a social behavior, male approach, which we detected using a set of pose-derived features from both social partners (the male must be close to the female, behind her and oriented toward her).

By aligning OE to the time of male approach, we find that our closed-loop control system can reliably trigger OE in virgin female flies with a total latency of 326 ± 150 ms (mean ± s.d.; Fig. 6f and Supplementary Video 5). Of this time, 77 ± 11 ms represents the system latency, measured by monitoring the onset of the optogenetic stimulus, and we estimate a biological latency (time from optogenetic stimulation to OE) of 249 ± 148 ms (Fig. 6g,h). We further show that OE is not observed following male approach in wild-type (WT) virgin females (Fig. 6f). These proof-of-principle experiments demonstrate that SLEAP can be used for online detection of social behaviors (here, male approach) in optogenetic perturbation experiments.

## Discussion

Here we have presented SLEAP, a general-purpose deep learning system for multi-animal pose tracking. This method advances the state of the art for both single-animal and multi-animal pose estimation and implements these innovations within a flexible and performant open-source framework designed for and tested by non-technical practitioners. SLEAP was built using industry-standard best practices in both software engineering and machine learning system design^27^. The modular construction of the subcomponents of SLEAP makes it easy to identify the source of errors or poor performance, which can then guide adjustments to the data-collection process and experimental design. In addition, we expose SLEAP’s modular functionality through documented APIs, which can be flexibly adopted in other frameworks^15^, and provide data-export formats to enable portability of SLEAP’s outputs for use in downstream analysis frameworks such as SimBA^14^ and B-SOiD^16^.

SLEAP’s modular design ensures that it is flexible. We find that SLEAP’s modular UNet architecture enables a ‘specialist’ paradigm in which small, lightweight models have just enough representational capacity to generalize to the low variability typically found in scientific data. This contrasts with the ‘generalist’ approach of training a single model that works on all datasets, a substantially harder task that comes as the cost of additional compute resource requirements and sacrifices accuracy within narrow domains (such as laboratory data) for generalizability in a broader domain. Indeed, without sacrificing accuracy, we observe large gains in performance, up to 11 times faster than the core network architecture used in DeepLabCut^5^. Additionally, by developing high-performance GPU implementations of core algorithms and leveraging state-of-the-art inference libraries, we achieve a latency four times lower than that reported in the DLCLive! package^24^, which was designed explicitly for real-time applications. Nonetheless, bridging the gap between specialist-level accuracy with generalist-level capacity remains an open problem in machine learning, which future work may seek to address in the domain of animal pose tracking. The dataset provided here (15,441 animal instances over 7,631 labeled frames and 337 trained models) should facilitate future developments.

Future work leading to the creation of new model types and approaches will be used to further improve SLEAP’s capabilities, including better incorporation of temporal information for more consistent tracking over time, alignment across multiple camera views to enable three-dimensional pose tracking and emerging techniques for self-supervised learning to improve the sample efficiency and generalizability of SLEAP on new datasets. The extensive documentation and software engineering practices employed in the development of SLEAP will facilitate these advances and serve as a resource for tool builders and practitioners alike.

## Methods

### Datasets

To evaluate how SLEAP performs across species, imaging conditions, experimental conditions and other properties of behavioral data that may affect pose-tracking performance, we built a collection of diverse animal pose datasets (Supplementary Table 1 and Supplementary Video 1). These data were manually labeled using the SLEAP labeling workflow (Supplementary Video 2). Below we describe in detail the seven datasets that we used in our analyses, which constitute a total of 7,636 labeled images with 15,441 animal instances. See the Supplementary Note for further description of the technical motivations for inclusion of each dataset.

To encourage further development and to provide the means for reproducible benchmarking of animal pose-tracking tools, we make these datasets available together with the images and training, validation and test set splits to ensure that new models are directly comparable with SLEAP. The 14 GB of labeled data are available in ref. ^28^.

#### Single fly

The ‘fly32’ dataset is a single-animal dataset that has been previously described and annotated with poses^6,29^. Here we use it for evaluating part-localization accuracy and inference performance for comparison with existing methods^5–7^.

Briefly, this dataset consists of 59 videos of a freely moving adult fruit fly (*Drosophila melanogaster*). During acquisition, a camera followed the animal in an arena 100 mm in diameter in real time; therefore, all frames are roughly centered on the fly. The chamber was backlit to provide maximum contrast; however, details on the top of the animal are not visible. Videos were recorded at 100 FPS with a resolution of 35 pixels per mm with a frame size of 192 × 192 × 1 in grayscale at a resolution of 35 pixels per mm.

For this dataset, we used the existing manual labels for a 32-node skeleton split into 1,200 training, 150 validation and 150 test frames^6^. No body parts are marked as not visible even when they are occluded; therefore, models trained on this dataset will be forced to ‘hallucinate’ missing body parts.

#### Flies

The ‘flies13’ dataset consists of 30 videos of freely interacting pairs of virgin male and female fruit flies (strain NM91) 3–5 d after eclosion. The animals were allowed to engage in courtship for up to 30 min or until copulation within a custom-fabricated behavioral monitoring chamber consisting of a 30-mm × 30-mm three-dimensional printed base (Formlabs Form 2, Black V3), a clear PETG vacuum-moulded dome (WidgetWorks Unlimited), a Blackfly S 13YM3-M USB3 camera (FLIR), an MVL35M23 35-mm FL C-mount lens (Thorlabs), a 25-mm premium 850-nm longpass filter (Thorlabs, FELH0850) and 850-nm IR LED strips for side illumination. The arena floor has visible microphone inlets for recording acoustic signals (not used in this study). The acquisition computers were custom-built workstations with Intel i7-8700K central processing units (CPUs), 64 GB of RAM, a Samsung 860 Evo Series 4-TB SSD for data and an EVGA GeForce GTX 1080 Ti 11-GB GPU. Videos were recorded from above at 150 FPS with an exposure time of 5 ms and a frame size of 1,024 × 1,024 × 1 and a resolution of 30.3 pixels per mm. Images were compressed in real time using Motif acquisition software (Loopbio) using GPU-accelerated H264 encoding with the ‘superfast’ preset of the libx264 library, resulting in nearly lossless videos with independently seekable frames.

We also collected but did not label videos in this behavioral monitoring setup with one, three, four and eight flies for inference speed benchmarking with variable number of animals.

For this dataset, we labeled 2,000 frames (4,000 instances) with a skeleton consisting of 13 nodes spanning clearly visible anatomical landmarks: head, thorax, abdomen, ‘wingL’, ‘wingR’, ‘forelegL4’, ‘forelegR4’, ‘midlegL4’, ‘midlegR4’, ‘hindlegL4‘, ‘hindlegR4’, ‘eyeL’ and ‘eyeR’; and 12 edges: thorax to head, thorax to abdomen, thorax to ‘wingL’, thorax to ‘wingR’, thorax to ‘forelegL4’, thorax to ‘forelegR4’, thorax to ‘midlegL4’, thorax to ‘midlegR4’, thorax to ‘hindlegL4’, thorax to ‘hindlegR4’, head to ‘eyeL’ and head to ‘eyeR’. Labels were randomly split into 1,600 training, 200 validation and 200 test frames. Additionally, for this dataset, we labeled each instance with the animal’s identity class as either ‘female’ or ‘male’ to enable ID model training.

For the closed-loop experiment, we generated an additional dataset (‘flies17’) in the same behavioral monitoring setup with an extended skeleton that included four additional nodes: ‘ovipositortip’, proboscis, ‘antennaeL’ and ‘antennaeR’. This smaller dataset (428 frames, 851 instances) was only used for the closed-loop analysis as it included the ‘ovipositortip’ landmark needed to measure OEs. To drive OE, we used a CsChrimson-expressing *GAL4* split: DNp13-SS2 (SS61090; gift from B. Dickson (Janelia Research Campus); full genotype, 20xUAS-csChrimson/VT038159.AD;VT029317.DBD/Sb).

#### Bees

The ‘bees’ dataset consisted of 18 videos of pairs of female worker bumblebees (*Bombus impatiens*) freely interacting in a Petri dish with hexagonal beeswax flooring for up to 30 min. The videos were recorded from above at 100 FPS with a frame size of 2,048 × 1,536 × 1 in grayscale at a resolution of 14 pixels per mm.

Queenright colonies of common eastern bumblebees (*B. impatiens*, *n* = 7) were purchased from Koppert Biological Systems between June and September 2019. Colonies were maintained in their original packaging under red light in a room with an ambient temperature of 74 °F. Bees were fed ad libitum on commercial sugar water (Koppert, 1.9-l bag per colony). All bees used for this dataset were 10 d old (10 d after eclosion).

For this dataset, we labeled 804 frames (1,604 instances) with a skeleton consisting of 21 nodes: ‘thor’, head, ‘abdo’, ‘Lant1’, ‘Lant2’, ‘Rant1’, ‘Rant2’, ‘fLleg1’, ‘fLleg2’, ‘fRleg1’, ‘fRleg2’, ‘mLleg1’, ‘mLleg2’, ‘mRleg1’, ‘mRleg2’, ‘hLleg1’, ‘hLleg2’, ‘hRleg1’, ‘hRleg2’, ‘Lwing’ and ‘Rwing’; and 20 edges: ‘thor’ to head, ‘thor’ to ‘abdo’, head to ‘Lant1’, head to ‘Rant1’, ‘Lant1’ to ‘Lant2’, ‘Rant1’ to ‘Rant2’, ‘thor’ to ‘fLleg1’, ‘fLleg1’ to ‘fLleg2’, ‘thor’ to ‘fRleg1’, ‘fRleg1’ to ‘fRleg2’, ‘thor’ to ‘mLleg1’, ‘mLleg1’ to ‘mLleg2’, ‘thor’ to ‘mRleg1’, ‘mRleg1’ to ‘mRleg2’, ‘thor’ to ‘hLleg1’, ‘thor’ to ‘hRleg1’, ‘hLleg1’ to ‘hLleg2’, ‘hRleg1’ to ‘hRleg2’, ‘thor’ to ‘Lwing’ and ‘thor’ to ‘Rwing’. Labels were randomly split into 642 training, 81 validation and 81 test frames.

#### Mice (home cage)

The ‘mice_hc’ dataset was used to evaluate performance under challenging imaging conditions with low contrast and a naturalistic setting. The dataset consisted of 40 videos of pairs of male and female 16-week-old white Swiss Webster mice (*Mus musculus*). Animals freely interacted for 5 min in a home cage environment with bedding to encourage naturalistic courtship behavior. The videos were recorded from above at 40 FPS with a frame size of 1,280 × 1,024 × 1 in grayscale using infrared illumination and a Blackfly Mono S camera (model BFS-US-13Y3M-C) at a resolution of 1.9 pixels per mm.

Experimental procedures were approved by the Princeton University Institutional Animal Care and Use Committee and conducted in accordance with the National Institutes of Health guidelines for the humane care and use of laboratory animals. Mice used in this dataset were purchased from Taconic Biosciences and had at least 1 week of acclimation to the Princeton Neuroscience Institute vivarium before experimental procedures were performed. Mice were co-housed with food and water ad libitum under a reversed 12-h–12-h dark–light cycle (light, 22:00–10:00).

For this dataset, we labeled 1,474 frames (2,948 instances) with a skeleton consisting of five nodes: snout, ‘earL’, ‘earR’, ‘trtb’ (tail base) and ‘tt’ (tail tip); and four edges: snout to ‘earL’, snout to ‘earR’, snout to ‘tb’ and ‘tb’ to ‘tt’. Labels were randomly split into 1,178 training, 148 validation and 148 test frames. We chose not to label the legs or paws because they were very rarely visible from a single top–down camera.

#### Mice (open field)

The ‘mice_of’ dataset was used to evaluate performance for tracking mice under optimal imaging conditions (high contrast) and with variable numbers of animals. The dataset consisted of videos from C57BL/6J male (*n* = 17) and female (*n* = 20) mice acquired from Jackson Laboratory (RRID:IMSR_JAX:000664, Jackson Laboratory). Groups of four and five mice were formed from same-sex littermates, and groups of two same-sex mice were picked randomly from different litters and interacted with each other in the open field for the first time. During video recording, mice moved freely in a 45.7 × 45.7-cm open-field arena with a clear acrylic floor. Videos were captured from below with infrared illumination using a Point Grey Blackfly S camera at a resolution of 1.97 pixels per mm at 80 FPS.

Experimental procedures were approved by the Princeton University Institutional Animal Care and Use Committee and conducted in accordance with the National Institutes of Health guidelines for the humane care and use of laboratory animals. Mice used in this study had at least 1 week of acclimation to the Princeton Neuroscience Institute vivarium in group cages with food and water ad libitum under a reversed 12-h–12-h dark–light cycle (light, 19:30–07:30) and were habituated in the dark test room for at least 30 min before experimental procedures were performed.

For this dataset, we labeled 1,000 frames (2,950 instances) with a skeleton consisting of 11 nodes: nose, neck, ‘L_ear’, ‘R_ear’, ‘L_Fr_paw’, ‘R_Fr_paw’, ‘tail_base’, ‘L_Hi_paw’, ‘R_Hi_paw’, ‘tail_mid’ and ‘tail_end’; and ten edges: neck to ‘L_Fr_paw’, neck to ‘R_Fr_paw’, ‘tail_base’ to ‘R_Hi_paw’, ‘tail_base’ to ‘L_Hi_paw’, ‘tail_base’ to ‘tail_mid’, ‘tail_mid’ to ‘tail_end’, neck to nose, neck to ‘R_ear’, neck to ‘L_ear’ and ‘tail_base’ to neck. Labels were randomly split into 800 training, 100 validation and 100 test frames.

#### Gerbils

The ‘gerbils’ dataset consisted of 23 selected videos from a continuous monitoring setup of a home cage with two pup and two adult gerbils (*Meriones unguiculatus*) from P15 to 8 months old. Animals lived freely in an open-field cage of approximately 60 cm × 40 cm with a clear acrylic floor for 20 d. Videos were captured continuously from above during white light and infrared illumination using a Point Grey Blackfly S camera at a resolution of approximately 2 pixels per mm at 25 FPS, totaling over 40 million frames over the 20-d study. Experimental procedures were approved by the New York University Institutional Animal Care and Use Committee and conducted in accordance with the National Institutes of Health guidelines for the humane care and use of laboratory animals. Gerbils used in this study were obtained from Charles River. Gerbils were kept in group cages with food and water ad libitum under a normal 12-h–12-h light–dark cycle (light, 07:00–18:00).

For this dataset, we labeled 425 frames (1,588 instances) with a skeleton consisting of 14 nodes: nose, left eye, right eye, left ear, right ear, ‘spine1’, ‘spine2’, ‘spine3’, ‘spine4’, ‘spine5’, ‘tail1’, ‘tail2’, ‘tail3’ and ‘tail4’; and 13 edges: ‘spine3’ to ‘spine2’, ‘spine2’ to ‘spine1’, ‘spine1’ to left eye, ‘spine1’ to left ear, ‘spine1’ to nose, ‘spine1’ to right eye, ‘spine1’ to right ear, ‘spine3’ to ‘spine4’, ‘spine4’ to ‘spine5’, ‘spine5’ to ‘tail1’, ‘tail1’ to ‘tail2’, ‘tail2’ to ‘tail3’ and ‘tail3’ to ‘tail4’. Labels were randomly split into 340 training, 43 validation and 42 test frames for training, validation and testing, respectively. Additionally, for this dataset, we labeled each instance with the animal’s identity class as female, male, ‘pupshaved’ or ‘pupunshaved’ to enable ID model training.

### Framework

Below, we describe the submodules that enable SLEAP’s functionality and outline the engineering considerations that were required in its design.

#### Labeling workflow

The SLEAP labeling workflow is enabled by its sophisticated user interface, allowing for consistent labeling practices to minimize differences across annotators and to facilitate user experience. This workflow is described in the Supplementary Note, illustrated in Supplementary Video 2 and described in the online documentation (https://sleap.ai).

#### Data model

SLEAP implements a comprehensive data model for describing and storing multi-animal pose-tracking data, for which no standard format has yet been described. In particular, we designed our data model with the goal of mitigating the risk of data dependencies in complex machine learning systems^27^ as well as to promote reproducibility and FAIR scientific data-stewardship best practices^30^.

To decouple a specific implementation of this data model from a logical schema that can be adapted without imposing a new software dependency, we describe a set of data structures that address two distinct needs in multi-animal pose tracking: training data and predictions. We provide a detailed description in Supplementary Table 3.

#### Model configuration

One of the design principles in SLEAP architecture was to decouple the configuration of training and inference jobs from their actual implementation(s). Consequently, SLEAP can import and export all user-controlled configuration parameters as standalone configuration dictionaries that are serializable to plain JSON files. These configuration files specify all the parameters required to run a training job or to perform inference from a trained model. Parameter specification is carried out through simple attributes that can be read and edited by a human as well as edited in a dedicated configuration GUI.

Decoupling configuration from implementation enables clean experimentation and hyperparameter tuning as well as convenient sharing of model training configurations (along with datasets) for reproducibility of results. See ref. ^28^ for our publicly available repository with 337 models (approximately 90 GB) that were trained over the course of this paper with various configurations.

#### Development and operations

**Testing and code quality** The test coverage in the SLEAP codebase is approximately 60%. We perform static type checking with MyPy (https://mypy.readthedocs.io/), and we automate code formatting with Black (https://black.readthedocs.io/).

**Packaging and distribution** SLEAP is automatically built and packaged on every merge to one of the primary branches (main, develop) using GitHub Actions. Upon release, the packages are published on PyPI and Conda package repositories. For PyPI distributions, users can configure GPU support on their own on any OS, while Conda distributions come with automatic GPU support for Windows and Linux.

**Deployment and execution options** SLEAP can be installed and executed locally on Windows, Linux or macOS (with or without GPU support) both in GUI or CLI modes. For remote execution (use cases include batch training or inference or access to a remote workstation with GPU support), SLEAP can be operated through a CLI as well as a high-level programmatic interface in Python. In particular, SLEAP training and inference can be executed in a Google Colab notebook with GPU support after exporting the labeled data package from the user’s local installation (one-click operation from the GUI).

**Documentation** A dedicated documentation website (https://sleap.ai) contains extensive documentation for end users as well as developers. Specifically, it provides a high-level overview of the main workflows, end-to-end tutorials with screenshots, detailed guides for specific features and tools, workshop videos, example data and sample training, inference and analysis code ready to launch in Google Colab with one click. For developers interested in contributing to SLEAP, the codebase is documented in detail throughout with Google-style docstrings.

#### Software stack

For the current version of SLEAP (version 1.1.4), the software stack consists of

- Graphics: PySide2 (5.14.1), Matplotlib (3.3.3)
- Interprocess communication: PyZMQ (20.0.0)
- Media input/output: FFmpeg (4.2.3), imgstore (0.2.9), scikit-video (1.1.11)
- Data models and serialization: attrs (19.3.0), cattrs (1.0.0rc0), h5py (2.10.0), jsmin (2.2.2)
- Numerical analysis: TensorFlow (2.3.1), imgaug (0.3.0), NumPy (1.18.5), OpenCV-Python (4.2.0.34)
- Engines: CUDA Toolkit (10.1.243), cuDNN (7.6.5), TensorRT (7.2.3.4).

For ease of distribution, we package several of our core dependencies (TensorFlow, NumPy, OpenCV, PySide2) as Conda packages for Windows and Linux. These are available at https://github.com/talmo/conda_packages.

We manage this software stack through Python buildtools-based requirements.txt as well as Conda environments. At every modification to the codebase, continuous integration enables verification of these dependencies through automated builds and tests on both Windows and Linux.

### Network architectures

SLEAP supports a large number of modular neural network architectures that are compatible with all approaches that we have implemented for multi-animal pose-tracking tasks. To standardize the configuration of these architectures and support exploratory research into the performance of different properties of neural networks, we describe all our models in terms of an encoder–decoder framework (Fig. 4a). This architectural motif was popularized by early work on image segmentation^31,32^, a task closely related to pose estimation in its construction (dense prediction). Namely, by leveraging fully convolutional architectures, we reduce the large space of hyperparameters of possible network instantiations to simple arrangements of a few blocks of layers that control high-level properties that can be easily explored:

1. Convolutional blocks are composed of one or more simple two-dimensional (2D) convolutional layers. We use a kernel size of 3 with a stride of 1 and ReLU activation for all layers. The main hyperparameter that we tune in this block is the number of filters in each block (16 to 64), which is secondarily controlled by a growth rate across sequential blocks (1.5 or 2). Increasing the number of filters affords the network greater representational capacity to learn more complex features at the cost of more parameters, which increase memory usage and computations.
2. Downsampling blocks are composed of a convolutional block that provides input to a two-strided maximum pooling layer with a kernel size of 3. Adding more downsampling blocks increases the maximum receptive field of the network and affords it the ability to integrate over larger-scale image features at the cost of more parameters and a loss in spatial resolution.
3. Upsampling blocks are composed of a convolutional block that provides input into either a transposed convolutional layer or a bilinear interpolant. We always use a stride of 2 when upsampling. These layers may be followed by additional 2D convolutional layers for refinement, a configuration that is more efficient when combined with bilinear upsampling than when using transposed convolutions alone. These blocks recover spatial resolution lost in the downsampling blocks but increase the memory and computations required as feature maps get larger. We typically use enough upsampling blocks to ensure an output stride of 2–4, that is, 1–2 fewer upsampling blocks than downsampling blocks.
4. Skip connections are topological features of fully convolutional architectures as they help to recover details from early downsampling blocks in the encoder by directly fusing these features with the correspondingly sized feature maps in the decoder. We employ skip connections for all variants of our models, including pretrained backbones by fusing intermediate-layer activations through simple addition of feature maps, possibly preceded by a 1 × 1 linear convolution to adjust the number of filters if they differ.

These basic components can be arranged to form the original network architecture that we employed in LEAP^6^ as well as modular UNet-like architectures^21^, which we now use as a default in SLEAP. Despite the simplicity of their design, our empirical evaluations have demonstrated that properties such as the RF of a model can be used to outperform even state-of-the-art pretrained network architectures with many times the number of parameters (Fig. 4f).

To calculate the maximum RF for a specific architecture, we use a closed-form equation for general convolutional architectures that can be derived from the configuration of the layers in these blocks^20^:

1 $${\textrm{RF}}=1+\mathop{\sum }\limits_{i=1}^{L}\left[({K}_{i}-1)\mathop{\prod }\limits_{j=1}^{i}{S}_{j}\right],$$

where *L* is the total number of layers in the encoder part of the network, *K*_*i*_ is the size of the convolutional kernel for the *i*th layer and *S*_*j*_ is the stride for the *j*th layer.

To compare configurations of our modular UNet to those of commonly used neural network architectures, we adapted a minimal open-source implementation of 32 reference architectures typically used for fully convolutional tasks such as semantic segmentation (https://github.com/qubvel/segmentation_models). This includes the ResNet, DenseNet, MobileNet, VGG, Inception and EfficientNet families of architectures and their variants, in addition to ImageNet-pretrained weights.

As described in more detail in Comparisons with DeepLabCut, we also implemented a faithful reproduction of the TF-Slim (https://github.com/google-research/tf-slim) version of ResNet. In particular, we developed extensive unit and integration tests with layer-by-layer and pretrained weight checksum comparisons. Additionally, a particularly relevant feature of the TF-Slim variant is the ability to adaptively modify select convolutional layers to use dilated (atrous) convolutions, which afford improved feature resolution typically lost in the encoder.

All these network architectures can be instantiated through SLEAP’s standardized configuration system, which is serializable into JSON files and stored with every trained model to enable reproduction of training configurations. We provide all model weights, training logs, configuration files and evaluation metrics for over 300 models (more than 90 GB) used in this paper in the associated repository^28^.

### Model training

Production-scale deep learning typically requires a large amount of expensive hardware to accelerate model training through the use of many GPUs or TPUs. Because the average practitioner typically only has access to a workstation equipped with consumer-grade GPUs, we designed our training procedures for high performance on single-GPU machines with relatively limited memory and computational resources. In addition to developing network architectures that can be configured with 10–100× fewer parameters than the more commonplace larger architectures, we also implemented a data pipeline system using performant CPU-parallelizable and GPU-parallelizable data loading, preprocessing, augmentation, shuffling, batching and caching by using the state-of-the-art tf.data system^33^.

We use a standard learning rate schedule with plateau detection for learning rate reduction and early stopping. We use the Adam optimizer with AMSGrad enabled and a default initial learning rate of 1 × 10^−4^. We use a mean squared error loss for all training targets except classification heads in ID models, in which case we use a cross-entropy loss. We train our models for 200 epochs at most, but very few training jobs reach this threshold before converging. We define an epoch as the total number of batches that are required to perform one iteration over the training dataset or 200, whichever is larger. At smaller sample sizes (10–100), this is compensated for by employing data shuffling and augmentation to reduce repeated training iterations on the same exact data samples. To maintain parity with most consumer-grade GPUs with limited memory, we use a batch size of 4 for all our training jobs. This enables model training even with high-resolution images such as the ‘bees’ dataset that has 1,536 × 2,048-pixel images; all other datasets are at least 1,024 × 1,024 pixels in size, which is often a minimum requirement to record animals with a large enough field of view without compromising on either experimental design or the spatial resolution required to resolve small body parts. For augmentation, we provide access to a wide variety of schemes through integration with the open-source imgaug library (https://github.com/aleju/imgaug); however, for all experiments described here, rotation is the only form of augmentation employed.

In the default SLEAP workflow, training and validation data splits are automatically generated with a ratio of 0.9 and 0.1, respectively, but, for all datasets in this paper, we pre-generated fixed training, validation and testing splits (ratios of 0.8, 0.1 and 0.1, respectively) to ensure reproducibility and fair comparisons of results across model runs. During the training procedure, SLEAP logs training and validation values for all loss terms for real-time monitoring and post hoc analysis. SLEAP publishes the training progress over a TCP port using ZeroMQ to communicate with the interactive training monitor provided in the GUI, which can also issue commands for manual early stopping of training jobs. SLEAP also generates visualizations of the raw outputs of the neural networks, such as confidence maps, evaluated on data sampled from the training and validation sets separately, to provide qualitative observations of training progress and the degree of overfitting. Additionally, losses and visualizations are saved to a disk in CSV form and the more detailed TensorBoard log format, which can optionally save performance-profiling data. Together, these features provide a rich source of diagnostic information for troubleshooting model performance and building intuition for dataset-specific nuances.

### Part localization

The position of each landmark from the labeled data is encoded for network training by a 2D array that we refer to as part confidence maps. For each body part coordinate ${{{{\bf{x}}}}}_{i}\in {{\mathbb{R}}}^{2}$, the value of the confidence map at pixel ${{{{\bf{x}}}}}_{\textrm{p}}\in {{\mathbb{R}}}^{2}$ is given by an unnormalized 2D Gaussian distribution,

2 $${{{{\bf{C}}}}}_{i}({{{{\bf{x}}}}}_{\textrm{p}})=\exp \left(-\frac{{\left\Vert {{{{\bf{x}}}}}_{i}-{{{{\bf{x}}}}}_{\textrm{p}}\right\Vert }_{2}^{2}}{2{\sigma }^{2}}\right){\delta }_{i},$$

where *σ* is a fixed scalar controlling the symmetric spread of the distribution and *δ*_*i*_ is equal to 0 when the body part is labeled as ‘not visible’ and equal to 1 otherwise.

The confidence maps are evaluated at each image grid pixel coordinate ${{{{\bf{x}}}}}_{\textrm{p}}\in \{\left((x,y)\right.:x\in \{0,...,W\},y\in \{0,...,H\}\}$, where *W* and *H* are the image width and height, respectively. The grid can be subsampled to generate lower-resolution confidence maps as targets for neural networks, trading off spatial resolution for decreased memory usage and compute cost. For an animal with *N* body part types (for example, head, thorax, etc.), we generate *N* confidence maps that are stacked along the channels axis such that the full confidence map’s tensor **C** is of shape (*H*/*s*_o_, *W*/*s*_o_, *N*), where *s*_o_ is the output stride of the network. Body parts that are marked as ‘not visible’ during labeling are represented by a confidence map filled with zeros. We set *σ* = 1.0 and scale by the output stride to maintain a fixed scale with respect to image resolution. For images with multiple instances of each body part type, the part confidence maps for each instance are combined by taking their maximum value at each pixel, which helps to separate closely spaced peaks^19^.

The confidence maps generated from the labeled data are used to train the neural network, which then predicts confidence maps for new data. The confidence map representation has the benefit of enabling fully convolutional neural network architectures that are both efficient and easier to train than networks that directly regress the coordinates of each body part^34^. The trade-off is that the coordinates must be computed from the confidence maps at inference time (that is, when the model is predicting new confidence maps).

For single-instance confidence maps, we decode the coordinates by finding the global peak, that is, the coordinates of the confidence map pixel with the highest value. For multi-instance confidence maps, we employ local peak finding, where we define a pixel as being a local peak if it is greater than its eight neighbors. In practice, we employ non-maximum suppression computed using a 2D grayscale dilation (maximum) filter with kernel

$${{{\bf{K}}}}=\left[\begin{array}{ccc}0&0&0\\ 0&-1&0\\ 0&0&0\end{array}\right].$$

**K** is convolved with the confidence map, producing a tensor whose elements contain the maximum of each 3 × 3 patch, excluding the central pixel. The pixels in the confidence map with values greater than those in the dilated maps are considered local peaks. In both global and local peak finding, we exclude peaks for which confidence map values fall below a fixed threshold, which we set to 0.2 to retain low-confidence predictions but exclude points that are definitively predicted as ‘not visible’.

As both these peak-finding methods can only yield peak coordinates at the resolution of the confidence map grid, localization accuracy is limited by the grid sampling interval. This quantization error is particularly problematic for models with larger output strides (that is, lower-resolution confidence maps); therefore, we employ subpixel refinement to improve peak coordinate localization. We leverage integral regression^35^ to compute real-valued offsets by taking the weighted average of the 5 × 5 local patch of the confidence maps around each grid-aligned peak and apply them to the coarser location estimates. We find that using *σ* values between 1.5 and 3.0 for the confidence maps is optimal for maximizing the performance of this subpixel-refinement step as larger values will result in confidence maps that are too broad.

### Bottom–up multi-animal pose estimation

For the bottom–up approach (Fig. 3a), we employ an image-based representation of the connectivity between body parts, called PAFs, that has been previously proposed for human pose estimation^19^. This representation captures the relationship between body parts explicitly by encoding a vector field that locally points from each source body part to each destination body part. This vector field is stored as two 2D images, one for each component in the *x*, *y* plane. To generate PAFs from labeled data, the user must define a directed graph, which we refer to as the skeleton, that connects all body parts to be tracked. In the bottom–up approach, a single neural network takes the full image as input and outputs both PAFs and multi-peak part confidence maps encoding the location of all body parts across all instances. By predicting both these representations, the network explicitly separates the task of localization and grouping, where, for one representation, it must only learn to predict ‘what’ a body part is (confidence maps), whereas, for the other, it must learn the relationship between them (PAFs). This is in contrast to the top–down approach, for which the relationship between body parts is implicitly encoded in the cropping. There are many possible skeletons that can be defined for a set of body parts, but, in practice, we find that optimal results are obtained when the depth of the skeleton graph is kept low (to reduce internode dependencies during matching) and the lines formed between the nodes actually overlap with the animal’s morphology in the image (making curved body parts such as rodent tails particularly challenging without intermediate keypoints).

A skeleton is defined as *S* = (*N*, *E*), where *N* is the set of *n* nodes (body parts) and *E* is the set of (*s*, *d*) tuples denoting the directed edge (connection) from a source node *s* ∈ {1,…, *n*} to a destination node *d* ∈ {1,…, *n*}/{*s*}. The direction at each point in the PAF derived from labeled data is generated from the coordinates of the body parts in labeled images by computing the distance-weighted edge unit vector for each edge *e* at each image grid coordinate **x**_p_,

3 $${{{{\bf{P}}}}}_{\textrm{e}}=\exp \left(-\frac{M({{{{\bf{x}}}}}_{\textrm{p}})}{2{\sigma }^{2}}\right){{{{\bf{u}}}}}_{\textrm{e}}{\delta }_{\textrm{s}}{\delta }_{\textrm{d}},$$

where **x**_s_ and **x**_d_ are the coordinates of the source and destination nodes, respectively. Similar to confidence maps, **x**_p_ may come from a subsampled image grid, *σ* controls the spatial spread of the PAF, *M* is the magnitude and *δ*_s_ and *δ*_d_ are the visibility flags for the source and destination nodes, respectively. The edge unit vector **u**_e_ is defined as the source-centered direction vector

4 $${{{{\bf{u}}}}}_{\textrm{e}}=({{{{\bf{x}}}}}_{\textrm{d}}-{{{{\bf{x}}}}}_{\textrm{s}})(\parallel {{{{\bf{x}}}}}_{\textrm{d}}-{{{{\bf{x}}}}}_{\textrm{s}}\parallel)^{-1} .$$

The magnitude *M* at each point in the PAF is defined as the Euclidean distance between the grid point **x**_p_ and its projection ${\hat{{{{\bf{x}}}}}}_{\textrm{p}}$ onto the line segment formed between **x**_s_ and **x**_d_, $M({{{{\bf{x}}}}}_{\textrm{p}})=\parallel {{{{\bf{x}}}}}_{\textrm{p}}-{\hat{{{{\bf{x}}}}}}_{\textrm{p}}{\parallel }_{2}$, where ${\hat{{{{\bf{x}}}}}}_{\textrm{p}}=r({{{{\bf{x}}}}}_{\textrm{d}}-{{{{\bf{x}}}}}_{\textrm{s}})+{{{{\bf{x}}}}}_{\textrm{s}}$ and

$$r=\min \left(\max \left(\frac{({{{{\bf{x}}}}}_{p}-{{{{\bf{x}}}}}_{s})\cdot ({{{{\bf{x}}}}}_{d}-{{{{\bf{x}}}}}_{s})}{\parallel {{{{\bf{x}}}}}_{d}-{{{{\bf{x}}}}}_{s}\underset{2}{\overset{2}{\parallel }}},0\right),1\right).$$

We note that the original description of PAFs^19^ uses a hard threshold to compute the distance weighting, but we adopt a Gaussian instead as a means of scaling the relative contribution of pixels as a function of distance from the edge, resulting in smoother PAFs with improved signal when animals are closely interacting as it reduces the effect of nearby opposing vectors canceling out their magnitudes.

PAFs computed for a given edge are combined for multiple instances by summation. After PAFs are generated for all edges in the skeleton, the full set of PAFs for the image **P** are of shape (*H*/*s*_o_, *W*/*s*_o_, 2∣*E*∣), formed by concatenating all individual edge PAFs, which contain the *x* and *y* components of the vectors along the third axis.

After confidence maps are converted to peaks via local peak detection, sets of candidate source and destination peaks are grouped via greedy bipartite matching using the PAFs to compute the score of each putative connection. For each pair of source and destination nodes, a line integral is computed by sampling ten evenly spaced points between source and destination coordinates in the predicted PAFs. The score for the connection is calculated as the average dot product between the sampled vectors (${\hat{{{{\bf{p}}}}}}_{\textrm{s}}$) and the unit normalized vector formed between the predicted source (${\hat{{{{\bf{x}}}}}}_{\textrm{s}}$) and destination points (${\hat{{{{\bf{x}}}}}}_{\textrm{d}}$) in the candidate connection,

5 $$\mathop{\sum }\limits_{s=1}^{10}\frac{{\hat{{{{\bf{x}}}}}}_{\textrm{d}}-{\hat{{{{\bf{x}}}}}}_{\textrm{s}}}{\parallel {\hat{{{{\bf{x}}}}}}_{\textrm{d}}-{\hat{{{{\bf{x}}}}}}_{\textrm{s}}{\parallel }_{2}}\cdot {\hat{{{{\bf{p}}}}}}_{\textrm{s}}.$$

Once all pairs of connections are scored, instances are assembled by growing their skeletons greedily edge by edge, assigning source candidates to destination candidates via Hungarian matching^36^. The detailed description of the instance-assembly algorithm and proof of its correctness can be found in the Supplementary Note.

### Top–down multi-animal pose estimation

In the top–down approach (Fig. 3b), each animal is first detected within the full-resolution image, and a bounding box is drawn around each animal to crop it from the frame. Each of the resulting crops will be centered on an animal but may contain pixels that belong to other animals. This centering is crucial as it provides spatial context to the second stage of the top–down approach, serving as an indicator of which animal’s body parts to predict within the cropped image. In our framework, we select a labeled body part type to use as an anchor, ideally one close to the center of the animal’s bounding box and infrequently occluded (if occluded, the centroid of the bounding box of the remaining parts is used as the anchor). The anchored part serves as the target for the first-stage neural network, which is trained to predict multi-peak confidence maps corresponding to the anchor part of all animals in the frame. Typically, this network is trained on downsampled full-frame images as coarsely locating the animals does not require high spatial resolution and saves on compute cost. Anchor part confidence maps are converted to coordinates using local peak finding and cropped from the full-resolution images with a fixed bounding box size computed automatically from the labels. In the second stage, we train a separate neural network that takes an instance-anchored image from the first stage and predicts single-peak confidence maps only for the anchored instance. The confidence maps are converted into coordinates using global peak finding as only a single set of body parts are expected. This network implicitly addresses the part-grouping problem by leveraging the location of the body parts relative to that of the anchor part (that is, the image center) as a cue for which body part to predict confidence maps for when multiples of the same body part type may be present within the crop. This form of implicit modeling of the geometry between body parts is simple and has been employed in animal pose literature previously^6,7^. The disadvantages of the top–down approach are that it fails to capture global contextual information present in the relationship between instances, is limited by the accuracy of the first-stage detector and requires a full forward pass through the second-stage network for each animal detected (although this may actually require less computation for images with few animals that occupy a small fraction of the image).

### Tracking

To address the problem of associating poses across frames, we devised a tracking algorithm that operates on grouped instances generated from the multi-animal pose estimation. The general tracking algorithm in SLEAP follows the standard multi-object tracking procedure. In brief, for each frame, we first generate a set of candidate instances from a window of recent frames that have been tracked, compute the matching cost between each candidate and each untracked instance in the current frame, perform optimal matching and assign them to tracks. For flow-shift-based tracking, we use optical flow^37^ to update past detection locations before computing the matching cost. See the Supplementary Note for a full description of the algorithm.

### Appearance-based ID models

Tracking-based approaches to maintaining animal identities consistent across frames are not well suited to long-term recordings or real-time applications due to the error propagation inherent in having temporal dependencies. To address these issues, we developed two approaches that rely on purely appearance-based features to simultaneously assign identities while detecting and grouping landmarks. We extended both forms of multi-instance pose-estimation approaches to address identification by casting it as a classification problem.

For bottom–up ID models, we modify the PAF-based approach by substituting PAFs with class segmentation maps. In this construction, we leverage the point–coordinate form of pose annotations to generate a binary mask at a fixed radius around each body part location. Masks for body parts are collapsed into distinct channels for each distinct animal class ID. We train this network with a cross-entropy loss for the class map head and mean square error for the multi-peak confidence maps, which are generated as in the conventional bottom–up approach. We note that, unlike the approach recently described in an update to DeepLabCut, we discard the PAFs in our ID models as they are redundant with the class maps. During inference, we group body parts via optimal matching of peaks to distinct classes based on the class map score sampled at the peak location. This bottom–up classification procedure implicitly groups body parts without requiring a superfluous skeleton model and an associated graph-parsing procedure.

For top–down ID models, we integrate conventional top–down pose estimation with simple classification of anchor-centered crops. Anchors are detected using the same first-stage network to generate locations for cropping. Next, we modify the centered-instance network to additionally predict class probabilities for the crop in the same fashion as previously described neural network-based identity-tracking approaches^8^. We project features from the deepest layer of the encoder into a 2D global maximum pooling layer, followed by a stack of three standard fully connected layers with 256 units and ReLU activations and finally a softmax layer to normalize the output logits. We train the classification head with a standard cross-entropy loss and weigh this loss term at a ratio of 0.0001 relative to the mean squared error term for the confidence maps. During inference, we aggregate classification probabilities for all crops in a frame and perform optimal matching between all candidate instances. This matching ensures that there are not more detections than classes and enforces mutually exclusive class assignments in addition to providing robustness to uncertainty in classifying individuals by leveraging probabilities from the others in the frame.

Importantly, we intuited that features from the deepest encoder layer carry considerably more information for resolving identities despite having poor spatial resolution. This is a well-known property of deep image-recognition networks from the extensive work in the image-classification domain in which a decoder is not typically employed to recover spatial resolution. We also posit this because confidence maps are a representation that are by construction disentangled from identity-specific features in favor of learning representations of morphological features that are invariant to variability in individual appearance. We empirically observed this behavior when we attempted to train top–down ID models using the penultimate decoder layer features instead of the deepest encoder layer features, resulting in extreme training instability and poor performance in both training targets.

### Inference models

During training, SLEAP models are saved with configuration metadata, and the neural network model architecture and weights are stored in HDF5-serialized SavedModel format only with the core native layers and operations in the high-level tf.keras API. This makes SLEAP models portable and free of external dependencies other than TensorFlow, but they do not include any custom operations necessary for inference.

During inference, SLEAP’s sleap.load_model() high-level API can be used to construct an inference model that wraps efficient, TensorFlow–AutoGraph-optimized^38^ data-preprocessing and prediction postprocessing routines (for example, peak finding, refinement and grouping) around the core model forward pass. Multi-stage models (that is, top–down) are assembled from individually trained submodels (for example, centroid and centered instance). Bottom–up model inference including PAF scoring, matching and grouping are largely reimplemented as GPU-native routines. Both local and global peak-finding operations as well as subpixel refinement are executed entirely on the GPU, thereby avoiding the bottleneck from transferring large tensors (that is, confidence maps) to the CPU. Once assembled, inference models can optionally be serialized as complete end-to-end SavedModels compatible with subsequent optimization and quantization using TensorRT. Post-training quantization into half-precision (FP16) models with TensorRT results in improvements in both inference throughput and latency but requires additional system configuration, which we describe in our extended installation instructions at https://sleap.ai.

### Accuracy metrics

To estimate the overall multi-animal accuracy, we calculated the object keypoint similarity (OKS) score, an accuracy metric that takes into account the uncertainty in landmark localization, visibility and animal scale^17^. To account for the uncertainty in landmarks that are difficult to label (such as subcutaneous landmarks), the OKS calculation uses a per-body part uncertainty factor derived from the variability across repeated labels of the same images by many individuals. Here, we set the uncertainty factor in our calculations to be equal to the least ambiguous human keypoint (eyes). This allowed us to interpret the OKS as a lower bound for the true accuracy of our models as some animal keypoints are challenging to locate precisely. We compute the OKS in its standard form:

6 $${\textrm{OKS}}({{{\bf{X}}}},\hat{{{{\bf{X}}}}})=\mathop{\sum }\limits_{i=1}^{N}\exp \left(-\frac{\parallel {X}_{i}-{\hat{X}}_{i}{\parallel }_{2}^{2}}{2\alpha {\sigma }_{i}^{2}}\right){\delta }_{i}(\mathop{\sum }\limits_{i=1}^{N}{\delta }_{i})^{-1},$$

where **X** and $\hat{{{{\bf{X}}}}}$ are the ground truth and predicted instance coordinates, respectively, for an instance with *N* nodes. *δ*_*i*_ denotes the visibility, which is 0 if the node is missing from the ground truth instance. The inner term essentially expresses the distance from the ground truth coordinate as the posterior of a Gaussian with two scaling terms: *α*, the bounding box area occupied by the GT instance, and *σ*_*i*_, the uncertainty factor (set to 0.025 for all measurements, equivalent to the uncertainty in labeling human eyes). Importantly, note the denominator, which indicates the count of visible body parts. In the case in which we have fewer body parts, such as in the ‘mice_hc’ dataset, having even a single missed body part will decrease the score considerably (for example, 4 ÷ 5 = 0.8) as compared to in the ‘mice_of’ dataset, which is labeled with more nodes (for example, 10 ÷ 11 = 0.91).

From the distribution of OKS scores, we derive the mAP as a summary of accuracy over the entire dataset. We adopt the same procedures as those employed in the widely used PoseTrack benchmark for human pose tracking^39^. Specifically, we compute the overall mAP using the same procedure employed for the PoseTrack benchmark and widely reported in human pose literature, a metric originally described in the Pascal visual object classes (VOC) challenge^40^. Briefly, mAP computation involves classifying each pairing of (greedily matched) GT and predicted instance as a true positive (TP) or false positive (FP) by using the OKS as a cutoff at each of the following thresholds: {0.50, 0.55, 0.60, 0.65, 0.70, 0.75, 0.80, 0.85, 0.90, 0.95}. Precision at a single threshold is calculated as TP/(TP + FP), and recall as TP/(TP + FN). All predictions are sorted by their OKS, and cumulative TPs and FPs are computed for each prediction, and recall and precision values are calculated from these partial TPs and FPs, that is, for each pair of GT and predicted instance. Next, a set of 101 recall thresholds are defined with even spacing from 0 to 1, and the best precision value for samples that fall below each recall threshold is retrieved from the data, yielding 101 precision values. The average precision is computed by taking the mean over all 101 precision values, whereas the average recall is defined as the best recall at the current OKS threshold. This procedure is repeated for all ten OKS thresholds, and the final mAP and mean average recall are the average of the average precision and average recall over all thresholds. Although their calculation is nontrivial, the mAP and mean average recall provide balanced estimates of consistently reliable precision and recall performance across many certainty thresholds.

For evaluating tracking performance, we leverage the open-source py-motmetrics (https://github.com/cheind/py-motmetrics) framework for multi-object-tracking benchmarking^41^. Here we primarily report on ID switches because it is the most actionable and relevant measure for practitioners as it directly quantifies the amount of proofreading labor required after tracking.

### Speed benchmarking

To evaluate model speed, we extracted 1,280-frames-long representative clips from each dataset. For the ‘flies13’ and ‘mice_of’ datasets, additional clips were created for videos with different numbers of animals to evaluate scaling with instance count.

To evaluate model inference performance, we first preloaded both the raw images for the entire clip and SLEAP-trained saved model weights. For top–down (centered-instance) models, we used the same dataset-specific centroid model. After loading, we performed one inference pass through the images to warm up the GPU and trigger AutoGraph tracing. In real-world scenarios, this warm-up cost is quickly amortized after the first few batches of data; therefore, we did not consider the first run in our timing measurements. Next, using the highest-resolution timer available on the system (PEP 418), we recorded the round-trip inference time, that is, the time elapsed between when a batch of images are accessed on the CPU to when results are received from the GPU and copied back to the CPU. This accounts for not only the model forward pass but also data transfer and other inference operations so as to reflect real-world performance. We repeated this procedure a minimum of three times and pooled the batch-wise results across all replicates.

All speed measurements were made on the same machine equipped with an Intel Core i7-10700K CPU, 64 GB of RAM and a Nvidia Titan RTX (24-GB) GPU running on Ubuntu 20.04 (64 bit). Unless otherwise noted, throughput (‘offline’) performance measurements were made with a batch size of 16 and TensorRT optimization (for top–down models, such as in Fig. 2).

### Experiments

#### Single-animal pose-estimation performance

To evaluate part-localization performance of each method independently of errors related to part grouping or identification, we used the ‘fly32’ dataset as it has been previously used to evaluate the performance of multiple pose-estimation tools. The large number of body parts, simple imaging conditions and large number of labels make it useful for establishing baseline performance in the optimal setting.

For DeepLabCut^5^, we fine tuned a ResNet50 with ImageNet-initialized weights. See Comparisons with DeepLabCut for more details on our implementation.

For DeepPoseKit^7^, we used the best DenseNet model trained on this dataset downloaded from the published repository at https://github.com/jgraving/DeepPoseKit-Data/blob/0aa5e3f5e8f9df63c48ba2bf491354472daa3e7e/datasets/fly/best_model_densenet.h5. We ran the native inference procedures in DeepPoseKit version 0.3.9.

For LEAP^6^, we trained a slightly modified version of the reference model described in the original paper, substituting transposed convolutions with bilinear interpolation for upsampling. We also used a higher-output stride to improve performance at the cost of confidence map resolution (in the original implementation, an output stride of 1 was used). We compensated for this loss of spatial resolution by using the subpixel-refinement routines that we implemented for SLEAP.

For SLEAP, we trained a UNet-based architecture on ‘fly32’ data. This is similar to the LEAP network but adds skip connections to recover spatial resolution in the feature maps of the decoder and uses a fewer number of filters per convolution.

Speed measurements were made as described in Speed benchmarking. Accuracy was measured using mAP as described in Accuracy metrics.

#### Multi-animal pose-estimation performance

Multi-animal pose-estimation speed and accuracy were evaluated as described in the above sections. For speed measurements in Fig. 2 (as related to batch size and number of animals), we used TensorRT-optimized versions of the best UNet-based SLEAP model for each dataset. For non-UNet architectures in subsequent analyses, we used the standard TensorFlow–AutoGraph inference models without TensorRT for speed measurements. Except for ‘best-model’-based analyses, we trained a minimum of three replicates of each model configuration in all experiments. Models that failed to converge within 200 epochs were excluded from subsequent analyses.

#### Sample efficiency

To estimate how accurate SLEAP models are when trained using different numbers of labeled frames, we generated labeled datasets with 5, 10, 20, 50, 100, 200, 300, 400, 500, 750, 1,000 and 1,500 frames sampled randomly from the training split of the ‘flies13’ and ‘mice_of’ datasets, which had the largest number of labels. The remaining held-out splits were kept fixed to ensure that accuracy was measured on the same test set.

#### Receptive field size

We evaluated the effectiveness of varying the maximum theoretical RF^20^ of our modular UNet network architecture as a mechanism for imposing an inductive bias toward relevant feature scales in models specialized to each dataset. We trained network configurations with two to seven downsampling blocks and adjusted the number of upsampling blocks to maintain a fixed output stride of 4 to control for spatial resolution of the outputs. We tested both top–down and bottom–up approaches and report the best of the two for each dataset. We conducted these experiments with the ‘flies13’, ‘mice_hc’ and ‘bees’ datasets to span a diversity of imaging conditions and anatomical feature scales.

#### Transfer learning

As the domain of animal pose estimation lacks large-scale labeled datasets for every species, imaging and experimental conditions, transfer learning has been proposed as an approach for reducing the need for labeled data^5^. To test this idea in the multi-animal setting, we trained top–down models on the ‘flies13’ dataset using 33 commonly used state-of-the-art network architectures as the encoder backbone with skip connections to a standard upsampling stack (bilinear interpolation, two refinement convolutions with 256 filters each, output stride of 4). Weights for the encoder were initialized either randomly or using ImageNet-pretrained weights. At least three replicates were trained for each encoder architecture and weight initialization approach; however, some architectures failed to converge entirely and were excluded from the analysis, although this may be addressed with further optimization hyperparameter tuning such as higher initial learning rates or additional training time. To measure the inference speed of these models, we applied our speed benchmarking procedure to a subset of the trained models (MobileNetV1, EfficientNetB0, SEResNet101, EfficientNetB7, VGG16 and ResNet50). For the remaining models, and to guide future network architecture configuration, we counted the number of computations (FLOPS) required to perform a forward pass of one image through the model and found that this static property of network architectures was highly correlated with real inference speed (Supplementary Fig. 4c).

#### Comparisons with DeepLabCut

To precisely implement the TF-Slim (https://github.com/google-research/tf-slim) version of ResNet used in DeepLabCut, we set the stride of the deepest convolution block to 1 to retain a higher-resolution output feature map with a stride of 16 and increased the block’s dilation rate to compensate for the decreased RF. For upsampling, we used nearly identical decoder architecture as the reference DeepLabCut implementation by stacking transposed convolutions with a stride of 2,256 filters and a kernel size of 4, followed by two regular convolutions for refinement. Their implementation, however, suffers from a loss of spatial resolution due to the repeated downsampling steps in the encoder; therefore, to encourage fairness in the comparisons, we added skip connections from the output of each downsampling block to the equivalently strided block in the decoder and fused the higher-resolution features through addition.

Our implementation of ResNet50 is more general and enables additional optimizations over that of DeepLabCut. The biggest architectural feature added in SLEAP’s version is the ability to use interpolation-based versus transposed convolution-based upsampling. We evaluated these two approaches on the ‘flies13’ and ‘mice_hc’ datasets and found that, while they achieve comparable accuracy (Supplementary Fig. 4d), interpolation-based upsampling considerably reduces the number of computations required.

We further compared both training and inference across ‘flies13’ and ‘mice_of’ datasets for convenient direct comparison of this implementation of the DLC ResNet and SLEAP’s UNet (Supplementary Fig. 5). These models use the same decoder for the ResNet and UNet, which is more compute efficient than the default DeepLabCut implementation.

Other network architectures used in DeepLabCut include MobileNet and EfficientNet, which we evaluated (in addition to dozens of others) in our transfer learning experiments.

The only other important difference between DeepLabCut and SLEAP’s low-level mechanics relates to subpixel localization of landmarks. DeepLabCut uses learnable refinement offset maps to regress more precise spatial coordinates than those afforded by their lower-resolution confidence maps. While SLEAP supports this functionality, we opted for an approach based on integral regression^35^ (see Part localization for details). We made this decision as integral regression is extremely fast at inference time and requires no additional loss term or costly optimization of an additional output target, thereby speeding up training and decreasing instability inherent in multi-task learning.

### Closed-loop control

Our closed-loop control system was built on a custom-fabricated behavioral monitoring chamber described earlier for the ‘flies13’ and ‘flies17’ datasets. For analog generation and acquisition, computers were equipped with a PCIe-6353 X Series DAQ board (National Instruments) interfaced with a BNC-2111 shielded connector block (National Instruments).

Custom experiment scripts for Motif software (version 0.1.9) were used for acquisition and GPU-accelerated compression and real-time streaming (Loopbio). Cameras were configured to trigger a 5-ms exposure at 150 FPS with 1,024 × 1,024-pixel frames, and videos were encoded with the ’superfast’ preset of the libx264 codec to ensure seekability.

For real-time control, custom scripts were written to query the image stream published by Motif during acquisition and perform online inference. SLEAP models were loaded and generated predictions on the latest image received from the camera in a separate thread from the acquisition and output generation. Using the detected poses, we classified whether the male was in an ‘approach’ pose based on the following criteria:

$$({\textrm{min}}\_{\textrm{dist}} < 2 {\textrm{mm}})\hspace{2.22144pt}{\textrm{and}}\hspace{2.22144pt}(| {\textrm{ang}}\,{{{\_}}}{\textrm{f}}{{{\_}}}{\textrm{rel}}{{{\_}}}\,{\textrm{m}}| < 2{5}^{\circ })\hspace{2.22144pt}{\textrm{and}}\hspace{2.22144pt}(| {\textrm{ang}}\,{{{\_}}}{\textrm{m}}{{{\_}}}{\textrm{rel}}{{{\_}}}\,{\textrm{f}}| > 14{5}^{\circ }),$$

where min_dist is the distance between the male’s head and the female’s abdomen tip, ang_f_rel_m is the angular location of the female thorax relative to the male’s heading and ang_m_rel_f is the angular location of the male thorax relative to the female’s heading. Together, these criteria will elicit a trigger condition when the male is behind the female, facing the female and within close proximity.

These criteria are evaluated in a separate thread every 25 ms, after which a 25-ms constant pulse of stimulation is output to the DAQ if the trigger condition was met or no stimulation voltage if it was not. These analog output signals were used to drive an array of 650-nm Luxeon Star LEDs to deliver optogenetic stimulation to the female. LEDs were positioned to achieve stimulation of roughly 100 μW mm^−2^.

For calibration experiments, we instead output the distance between the thorax of the male and female, scaled to 1.0–5.0 V and read back in through a loopback connection to an analog input on the same DAQ. These signals were then scaled back to physical units and aligned to offline tracking to estimate the system latency by using dynamic time warping on non-overlapping 1-s segments across the entire session.

### Reporting Summary

Further information on research design is available in the Nature Research Reporting Summary linked to this article.

## Online content

Any methods, additional references, Nature Research reporting summaries, source data, extended data, supplementary information, acknowledgements, peer review information; details of author contributions and competing interests; and statements of data and code availability are available at 10.1038/s41592-022-01426-1.

## Supplementary information

Supplementary Information Supplementary Tables 1 and 2 and Note

Reporting Summary

Supplementary Tables 3–5 Supplementary Tables 3–5 (workbook). Supplementary Table 3: Schema of data model developed for pose-estimation data. Supplementary Table 4: Table of main datasets used in the paper with metadata. Supplementary Table 5: Table of all models used in the paper with metadata.

Supplementary Video 1 Example predictions across all datasets. These are outputs from SLEAP that have not been proofread or corrected.

Supplementary Video 2 Screencast of a SLEAP labeling session following our recommended protocol and demonstrating GUI functionality.

Supplementary Video 3 Visualization and descriptions of the stages involved in bottom–up and top–down multi-instance pose-estimation approaches.

Supplementary Video 4 Comparison of temporal-based versus appearance-based approaches for assigning animal identities, highlighting the error-propagation behavior of the tracking method.

Supplementary Video 5 Example bout from our closed-loop social behavior-control system, illustrating the timescale at which SLEAP tracks poses and identities to detect behaviors and drive optogenetic stimulation to trigger a social behavior response.

## Data Availability

We provide all model weights, training logs, configuration files and evaluation metrics for over 300 models (more than 90 GB) used in this paper in the associated repository^28^. An overview of the datasets is provided in Supplementary Table 1. Best models for each dataset are summarized in Supplementary Table 2. Full metadata for each dataset are provided in Supplementary Table 4. All model metadata are provided in Supplementary Table 5, including which figures they are associated with. Source data are provided with this paper.

## Source data

Source Data Fig. 2 Source data for plots.
Source Data Fig. 3 Source data for plots.
Source Data Fig. 4 Source data for plots.
Source Data Fig. 5 Source data for plots.
Source Data Fig. 6 Source data for plots.
Source Data Extended Data Fig. 5 Source data for plots.
Source Data Extended Data Fig. 6 Source data for plots.

## Code availability

Our software is freely available as open-source software at https://github.com/murthylab/sleap. It can be installed via standard Python package repositories: conda install -c sleap -c conda-forge -c nvidia sleap or pip install sleap. Documentation, guides, notebooks, tutorials and more are available on the main website at https://sleap.ai. SLEAP is licensed under a modified BSD-3 license, which permits unrestricted usage for non-commercial applications. To ensure reproducibility of the analyses presented in all figures of this paper, we provide code to generate all non-schematic figure panels as a Code Ocean capsule.
