# Publisher Correction: SLEAP: A deep learning system for multi-animal pose tracking

**DOI:** 10.1038/s41592-022-01495-2

**Published:** 2022-04-25

**Authors:** Talmo D. Pereira, Nathaniel Tabris, Arie Matsliah, David M. Turner, Junyu Li, Shruthi Ravindranath, Eleni S. Papadoyannis, Edna Normand, David S. Deutsch, Z. Yan Wang, Grace C. McKenzie-Smith, Catalin C. Mitelut, Marielisa Diez Castro, John D’Uva, Mikhail Kislin, Dan H. Sanes, Sarah D. Kocher, Samuel S.-H. Wang, Annegret L. Falkner, Joshua W. Shaevitz, Mala Murthy

**Affiliations:** 1 Princeton Neuroscience Institute, Princeton University, Princeton, NJ USA; 2 Department of Molecular Biology, Princeton University, Princeton, NJ USA; 3 Department of Ecology and Evolutionary Biology, Princeton University, Princeton, NJ USA; 4 Lewis–Sigler Institute for Integrative Genomics, Princeton University, Princeton, NJ USA; 5 Department of Physics, Princeton University, Princeton, NJ USA; 6 Center for Neural Science, New York University, New York, NY USA; 7 Department of Psychology and Department of Biology, New York University, New York, NY USA; 8 Present Address: The Salk Institute for Biological Studies, La Jolla, CA USA; 9 Present Address: Department of Biomedical Engineering, Johns Hopkins University School of Medicine, Baltimore, MD USA

**Keywords:** Computational neuroscience, Software, Machine learning

Correction to: *Nature Methods* 10.1038/s41592-022-01426-1, published online 4 April 2022.

In the version of this article initially published, there were errors in the affiliations shown for John D’Uva. The correct affiliations should read: ^1^Princeton Neuroscience Institute, Princeton University, Princeton, NJ, USA; ^2^Department of Molecular Biology, Princeton University, Princeton, NJ, USA; and ^9^Present address: Department of Biomedical Engineering, Johns Hopkins University School of Medicine, Baltimore, MD, USA. Further, the source data files for Extended Data Figs. 5 and 6 were initially listed as source data for Extended Data Figs. 4 and 5. The files have been revised online and the errors corrected in the HTML and PDF versions of the article.

