# Supplementary material for: SLEAP: A deep learning system for multi-animal pose tracking
Source: Nat Methods. 2022 Apr 4;19(4):486–95. doi: 10.1038/s41592-022-01426-1 (PMC9007740; doi:10.1038/s41592-022-01426-1)
Supplement: Supplementary file 1 — Supplementary Tables 1 and 2 and Note [file 41592_2022_1426_MOESM1_ESM.pdf]

nature portfolio

https://doi.org/10.1038/s41592-022-01426-1

Supplementary information

SLEAP: A deep learning system for multi-animal pose tracking

In the format provided by the
authors and unedited

Supplementary Tables

Name	Videos	Image size	Resolution*	Parts	Labels	Split	Instances	Animals	ID
fly32	59	192 x 192 x 1	35	32	1500	1200/150/150	1500	1	✗
flies13	30	1024 x 1024 x 1	30.3	13	2000	1600/200/200	4000	2	✓
flies17	44	1024 x 1024 x 1	30.3	17	428	385/43/0	851	2	✗
bees	18	1536 x 2048 x 1	14	21	804	642/81/81	1604	1-2	✗
mice_hc	40	1024 x 1280 x 1	1.9	5	1474	1178/148/148	2948	2	✗
mice_of	20	1280 x 1024 x 1	1.97	11	1000	800/100/100	2950	1-5	✗
gerbils	23	1024 x 1280 x 3	1.5	14	425	340/43/42	1588	2-4	✓

Supplementary Table 1: Summary of all datasets used. See Supplementary Table 4 for full metadata. * Resolution values in units of pixels per mm.

Dataset	Approach	mAP	mAR	95% (px)	95% (mm)	ID (%)	Parameters (M)	GFLOPS
fly32	Single animal	0.927	0.937	3.61	0.10	nan	7.811	12.98
flies13	Top-down	0.825	0.879	3.18	0.11	99.8	1.686	1.90
bees	Bottom-up	0.679	0.709	16.78	1.20	nan	6.790	313.72
mice_hc	Bottom-up	0.513	0.570	16.20	8.52	nan	2.937	124.35
mice_of	Top-down	0.774	0.839	6.46	3.28	nan	2.936	11.76
gerbils	Bottom-up (ID)	0.316	0.430	19.50	13.00	92.4	6.786	131.98

Supplementary Table 2: Summary of best model for each dataset. See Supplementary Table 5 for full metadata.

Supplementary Protocol

The SLEAP interface supports a standardized and reproducible workflow for generating labeled data for training models on new datasets. Here we outline the protocol we asked labelers to follow (Supplementary Video 2):

1. Add videos of multiple sessions to your project. These should span the diversity of your data (days, animals, setups, experimental conditions, etc.). You can add more videos to the project later, and you also don't need to add every video that you'll be predicting on. A typical labeling dataset will have 10 to 30 sessions.
2. Create a skeleton. Edges can be changed later if needed for bottom-up models, so initially just connect the nodes however you prefer for ease of visualization.
3. Generate suggestions to build a labeling queue of frames that are representative of the diversity of image features in the data.
4. Do the first labeling round. Label 10 to 20 frames across suggestion groups and videos. Start with easy examples where the body parts are most visible and animals are in typical poses.
5. Run the first round of training. Model selection is highly data dependent, but the default top-down model configuration will work well for most cases. If training locally, predictions on unlabeled suggested frames will be automatically generated and merged into the project. If training remotely (e.g., on Colab or a compute cluster), download the resulting predictions (also stored in a `.slp` labels files) and merge them into the base project.
6. Save the labels as a new file to ensure that you have checkpoints that you can go back as you progress through the labeling process. In the GUI, selecting **File**, then **Save as...** will automatically increment the version number of your labels file for convenient checkpointing.
7. Sort the predicted suggestions by score to identify frames that the model has a hard time with or to find easy frames that increase the diversity of the labels. On frames with predictions, double click on each predicted instance to create a labeled instance that you can correct by adjusting nodes that are misplaced or missing altogether.
8. After labeling an additional 20 to 40 frames, retrain and import the new predictions, which should be considerably improved relative to the previous iteration. You will do this for multiple rounds until you begin to see negligible improvements with further labeling.

9. Once at 100 to 200 labeled frames, you can try to tweak the model configuration and explore different approaches, such as bottom-up versus top-down or different network architectures, and see what works best for your data or achieves acceptable accuracy with increased speed.
10. The final result of the labeling workflow is one or more trained models that achieve the desired level of accuracy on your data. You can use these to track the entire dataset, or entirely new videos. This can be done separately from the labeling project and predictions will be generated on a per-video basis. These predictions can be opened in the SLEAP GUI for inspection and tracking proofreading.
11. In the proofreading stage, the goal is to identify identity switches which propagate to subsequent frames. This can be done by visual inspection, or guided by a number of metrics that SLEAP can calculate for each frame such as average prediction score, maximum velocity and more.
12. After proofreading, the final results can be exported to a format convenient for analysis. SLEAP saves the tracking data (poses and identities) in an HDF5 format where predictions are stored in a contiguous array of shape $frames \times nodes \times xy \times animals$ containing the image coordinates of each body part for all animals and frames of a video. This can be read in without any additional dependencies in any language that supports HDF5 such as MATLAB, R or Python.

Supplementary Note

Dataset selection

`fly32`

This dataset was employed for comparison with existing pose estimation frameworks and to disentangle the improvements contained in SLEAP associated with its generalization to the multi-animal domain.

`flies13`

We use this dataset as our “gold standard” for social behavioral data as it was recorded at high resolution with optimal illumination, large frame size and high FPS. This permitted the exploration of a wide range of model configurations that must be fast despite the large image size (1024 x 1024) without sacrificing accuracy. This is particularly challenging as the resolution cannot be reduced without losing information about the thinnest body parts (leg tips), thereby precluding trivial solutions such as image downsampling. This dataset is also ideal for evaluating distinct approaches to identification as the animals are both subtly different in appearance and have many highly visible body parts that have low displacement and motion blur across frames due to the low exposure time, therefore making it compatible with either appearance- or temporal-based models.

`bees`

Similar to the fly dataset, this resolution is close to the minimum required in order to reliably capture the finer features of the bees such as their tarsi tips and antennae, both of which are frequently employed in social interactions [1]. This presented a distinct challenge as the large body size of the bees (200 to 400 pixels) together with the finest feature sizes (2 to 4 pixels) required models to simultaneously capture features across a wide range of length scales.

`mice_hc`

This dataset is representative of a naturalistic rodent social behavior paradigm as mice were allowed to interact freely within a familiar home cage environment. This setting presents challenging imaging conditions due to low contrast as a result of low-power IR illumination and white fur color of the animals against the bedding material. Additionally, the few body parts labeled and low FPS present a distinct challenge for identity tracking. Temporal association methods must be capable of simultaneously handling large displacements across frames, while also remaining robust to unreliable landmarks due to heavy occlusion due to social interactions, frequently resulting in as few as 1 to 3 body parts detected per animal within a frame.

`mice_of`

Unlike the home cage dataset, this dataset is imaged from below with high contrast, affording greater visibility of the paws during locomotion. This is representative of ideal imaging conditions, but comes at the cost of not being compatible with the use of bedding to promote a more ethologically relevant natural setting. The difference in the number of animals and the occasional use of head mounts make this dataset ideal for studying in-domain generalization

performance since it requires models to generalize across different experimental conditions, but *not* imaging conditions as they are all recorded in the same open field arena.

`gerbils`

This dataset presented a constellation of unique challenges that test the limits of multi-animal pose tracking methods. The imaging conditions suffer from heavy lens distortion, sub-optimal focus, low FPS with long exposure times resulting in considerable motion blur, highly variable illumination due to the light-dark cycle and the use of white light for illumination without filtering. The experimental conditions are challenging due to several factors: gerbils frequently engage in huddling behaviors resulting in heavy occlusion and making it difficult to identify individual animals or assign body parts to the correct one; the monitoring is done in a home cage environment with shifting bedding that has low contrast with the animals' fur, as well as enrichment objects which can occlude the animals from the camera during interactions; large variability in animal size due to the range of ages, requiring exceptional robustness to multi-scale image features; and most challenging of all: the videos are recorded continuously over the span of multiple days. Unlike the other datasets we used, which consist of sessions on the order of tens of minutes, this dataset makes it intractable to use temporal association-based models as even rare identity switching would make proofreading extremely laborious as errors would be difficult to identify and correct, in addition to propagating over millions of frames. This is further compounded by having 4 animals which considerably increases the number of possible incorrect combinations of identity assignments. This makes this dataset ideally suited for appearance-based ID models which can leverage the variability in body morphology and fur patterning across animals as distinguishing features, and which do not rely on temporal dependencies across frames thereby guaranteeing that ID errors will not be propagated over time which effectively eliminates the need for proofreading.

Tracking algorithm

Algorithm 1: Tracking algorithm

```
Result: Tracked instances
D ← {} // Tracked instance deque
for t ∈ {1, ..., T} do
    I_c ← Generate candidate instances from D
    I_t ← Grouped instances in frame t
    C ← Cost(I_c, I_t) // Pairwise costs
    M ← Find optimal matching
    for (i, j) ∈ M do
        AppendToTrack(Track(I_c[i]), I_t[j])
    end
    for k ∉ M do
        SpawnNewTrack(I_t[k])
    end
    AppendToDeque(I_t)
end
```

To adapt our tracking algorithm to the task of pose tracking specifically, we first employ flow shift for candidate generation. Inspired by previous work on multi-human pose tracking [2], the flow shift generator takes instances from previous frames and applies Farneback optical flow [3] to predict the displacement of the image between pairs of past frames and the current frame. We apply the displacements to the coordinates of past poses to generate a set of "shifted" instances with locations predicted by the image motion. This considerably improves the similarity between instances in the past and present ones, especially during bouts of fast social behaviors with large displacements across frames (e.g., chasing) during which the past location of one instance may more closely overlap with the current location of another. To compute the matching cost between instances, we use an unnormalized instance similarity score defined as

$$\exp\left(-\|I_c - I_j\|_2^2\right)/v_c, \tag{1}$$

where v_c is the number of visible landmarks in the candidate instance.

We note that this system can be extended to arbitrary candidate generation and matching score functions, such as learnable affinity matching or autoregressive motion models.

Bottom-up instance assembly

In this section we describe how animal instances are assembled in a bottom-up fashion from body-part confidence maps and their affinity scores. Let's start with definitions:

- **Skeleton** is a collection of n nodes (body parts) and m edges (connections between pairs of body parts). For the reasons described below, we impose a constraint on the underlying graph of nodes and edges to form a *tree*, which implies $m = n - 1$.
- **Peak** is a point in the image detected to match a specific node i from the skeleton. In a multi-instance setting each skeleton node is expected to have multiple peaks, denoted by $p_1^i, \ldots, p_K^i$ for every node $i \in \{1, \ldots, n\}$. Here K denotes the number of animal instances, and in this section we also assume that the peaks $p_1^i, \ldots, p_K^i$ for each node i were detected correctly by the neural network[1].
- **Affinity scores** are assigned to pairs of peaks along with a corresponding edge e from the skeleton. Specifically, for every edge $e = i \to j$ from the skeleton and every pair p_x^i, p_y^j of peaks, the PAF [4] model outputs a corresponding affinity score $s_{x,y}^{i,j}$, where higher affinity score is interpreted as higher likelihood of the peaks p_x^i, p_y^j belonging to the same instance[2].

Problem statement

With these definitions, the problem of assembling instances from detected peaks and affinity scores can be stated as follows:

- **Input:** Number of instances K, skeleton tree with n nodes and $m = n - 1$ edges, nK peaks, mK^2 affinity scores.
- **Output:** Partition the nK peaks into K disjoint sets $I_1, \ldots, I_K$ of size n each, so that for every group I_k and node i in the skeleton, there is exactly one peak p_*^i in I_k.
- **Objective:** Maximize the total sum of affinity scores for pairs of peaks *contained* within the same set in the partition $I_1, \ldots, I_K$.

Algorithm

As initially proposed in [4] we start by matching peaks along skeleton edges into disjoint pairs. Specifically, for every skeleton edge $e = i \to j$ we find K pairwise disjoint pairs of peaks $(p_1^i, p_1^j), \ldots, (p_K^i, p_K^j)$ so that the sum of the corresponding affinity scores is maximized. Finding the maximum-weight matching in the underlying weighted bipartite graph can be done efficiently using e.g. the Hungarian Maximum Matching Algorithm (time complexity $O(K^3)$).

Then we proceed to grouping the selected pairs of peaks into K instances. This is done by assembling connected components from nK nodes and mK edges (selected pairs of peaks from previous step) as follows:

1. partition the peaks into nK singleton sets;
2. for each edge corresponding to selected pair of peaks p, p', merge the sets to which p and p' belong into one.

Correctness

In every iteration from step 2, p and p' belong to different sets. This is because we selected the pairs of peaks according to the skeleton edges, and the skeleton is a tree (has no cycles). Hence, in every iteration the number of sets decreases by one.

Since we start with nK sets and perform $mK = (n-1)K$ iterations, the final number of sets is exactly K. Furthermore, by construction, each set contains exactly one peak for every node of the skeleton, as required.

Regarding the objective to maximize affinity scores contained within the partition sets, note that for all edges of the skeleton, the corresponding pairs of peaks are matched to maximize the sum of scores *independently of other skeleton edges* (here too using the fact that the underlying skeleton graph is a tree) . Therefore the total sum of affinity scores contained within the partition sets is maximized.

[1] Without such assumption, assembling detected peaks into instances correctly – the problem addressed in this section – would be impossible.

[2] In practice, pairs of peaks with affinity score lower than a certain threshold can be dismissed during the instance assembly phase for tractability.

Time complexity

Finding the maximum-weight matching for every edge in the skeleton takes $O(mK^3) = O(nK^3)$ time. In the grouping phase what we do is essentially finding connected components in an undirected graph of size $O(mK) = O(nK)$. This takes linear time, and so the total time complexity governed by the first phase, and it remains $O(nK^3)$.

Note on solving the general case

Finding the partition with the maximum total sum of affinity scores in the general case, when the skeleton is not constrained to be a tree, is an NP-hard problem (and so, unlikely to have a computationally efficient solution for inputs of non-trivial size). This is because the problem of finding such a partition is a generalization of the well-known 3-dimensional matching problem that is known to be NP-hard[3].

SLEAP Experimentation Framework

One of the main advantages of SLEAP's modular design is that it enables running large scale experiments and hyper-parameter tuning without having to alter its source code. Our setup for measuring and tuning the performance of SLEAP on large collections of data (or large variety of configurations) is depicted in Supplementary Note Figure 1. This is a distributed experimentation framework that scales well and handles hardware or network failures reliably. The

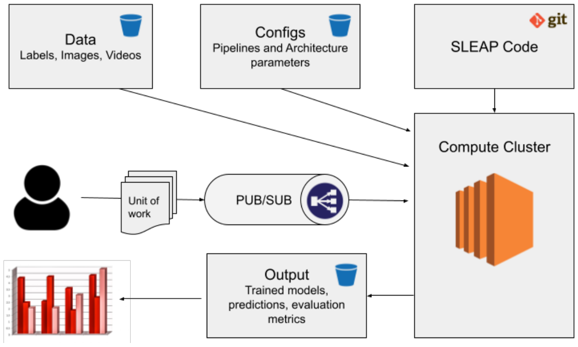

Figure 1: SLEAP Experimentation Framework

workflow follows these steps:

- User uploads the SLEAP packages (labels, raw-data and configuration files) to a shared bucket that the worker machines have access to.
- User posts a sequence of messages on a PUB/SUB system, each message consists of: experiment name, dataset location, configuration with preprocessing, training and inference params, and git SHA of the source of SLEAP to be used. With this information, each message corresponds to an idempotent unit of work that can be executed on any worker machine independently[4].
- User spins up N virtual machines from a pre-baked image, containing environments and scripts for processing messages, pulling and updating the version of SLEAP, fetching the relevant data and configuration files from the shared bucket, running SLEAP training, inference and tracking, and writing results to the specified experiment output location (for instance, cloud storage bucket).
- Each one of these VMs starts polling and processing messages as soon as it comes online. Once all the messages have been consumed and processed, the user shuts down the worker machines (if applicable) and downloads the generated output data for further analysis.

References

[1] Goulson, D. *Bumblebees: Behaviour, Ecology, and Conservation* (OUP Oxford, 2010).

[3] In fact, 3-dimensional matching problem is one of the original list of 21 problems from Karp's seminal work that started the study of computational complexity [5].

[4] The popular PUB/SUB services (e.g. on GCP or AWS) provide failure handling features, such as re-delivering un-acknowledged messages to a new worker after a configurable time period. This reduces the overhead of monitoring worker machines and recovering from failures significantly.

[2] Xiao, B., Wu, H. & Wei, Y. Simple baselines for human pose estimation and tracking. In *Proceedings of the European conference on computer vision (ECCV)*, 466–481 (2018).

[3] Farnebäck, G. Two-Frame motion estimation based on polynomial expansion. In *Image Analysis*, 363–370 (Springer Berlin Heidelberg, 2003).

[4] Cao, Z., Simon, T., Wei, S.-E. & Sheikh, Y. Realtime Multi-Person 2D pose estimation using part affinity fields. In *Proceedings of the IEEE conference on computer vision and pattern recognition*, 7291–7299 (2017).

[5] Karp, R. M. *Reducibility among Combinatorial Problems* (Springer, Boston, MA, 1972).
