# Supplementary material for: SLEAP: A deep learning system for multi-animal pose tracking
Source: Nat Methods. 2022 Apr 4;19(4):486–95. doi: 10.1038/s41592-022-01426-1 (PMC9007740; doi:10.1038/s41592-022-01426-1)
Supplement: Supplementary file 2 — Reporting Summary [file 41592_2022_1426_MOESM2_ESM.pdf]

Corresponding author(s): Mala Murthy

Last updated by author(s): Dec 1, 2021

Reporting Summary

Nature Research wishes to improve the reproducibility of the work that we publish. This form provides structure for consistency and transparency in reporting. For further information on Nature Research policies, see our Editorial Policies and the Editorial Policy Checklist.

Statistics

For all statistical analyses, confirm that the following items are present in the figure legend, table legend, main text, or Methods section.

n/a	Confirmed	
☐	☒	The exact sample size (*n*) for each experimental group/condition, given as a discrete number and unit of measurement
☐	☒	A statement on whether measurements were taken from distinct samples or whether the same sample was measured repeatedly
☒	☐	The statistical test(s) used AND whether they are one- or two-sided<br>*Only common tests should be described solely by name; describe more complex techniques in the Methods section.*
☒	☐	A description of all covariates tested
☒	☐	A description of any assumptions or corrections, such as tests of normality and adjustment for multiple comparisons
☐	☒	A full description of the statistical parameters including central tendency (e.g. means) or other basic estimates (e.g. regression coefficient) AND variation (e.g. standard deviation) or associated estimates of uncertainty (e.g. confidence intervals)
☒	☐	For null hypothesis testing, the test statistic (e.g. F, t, r) with confidence intervals, effect sizes, degrees of freedom and P value noted<br>*Give P values as exact values whenever suitable.*
☒	☐	For Bayesian analysis, information on the choice of priors and Markov chain Monte Carlo settings
☒	☐	For hierarchical and complex designs, identification of the appropriate level for tests and full reporting of outcomes
☒	☐	Estimates of effect sizes (e.g. Cohen's *d*, Pearson's *r*), indicating how they were calculated

Our web collection on statistics for biologists contains articles on many of the points above.

Software and code

Policy information about availability of computer code

Data collection	Loopbio Motif v0.1.9
Data analysis	SLEAP (1.1.4; https://github.com/murthylab/sleap), PySide2 (5.14.1), Matplotlib (3.3.3), pyzmq (20.0.0), ffmpeg (4.2.3), imgstore (0.2.9), scikit-video (1.1.11), attrs (19.3.0), cattrs (1.0.0rc0), h5py (2.10.0), jsmin (2.2.2), TensorFlow (2.3.1), imgaug (0.3.0), numpy (1.18.5), OpenCV-Python (4.2.0.34), CUDA Toolkit (10.1.243), CuDNN (7.6.5), TensorRT (7.2.3.4), DeepPoseKit (0.3.9).

For manuscripts utilizing custom algorithms or software that are central to the research but not yet described in published literature, software must be made available to editors and reviewers. We strongly encourage code deposition in a community repository (e.g. GitHub). See the Nature Research guidelines for submitting code & software for further information.

Data

Policy information about availability of data

All manuscripts must include a data availability statement. This statement should provide the following information, where applicable:

- Accession codes, unique identifiers, or web links for publicly available datasets
- A list of figures that have associated raw data
- A description of any restrictions on data availability

In order to encourage further development and to provide the means for reproducible benchmarking of animal pose tracking tools, we make these datasets available together with the images and training/validation/test set splits to ensure that new models are directly comparable with SLEAP. The 14 GB of data are available at: https://dx.doi.org/10.17605/OSF.IO/36HAR

We provide all model weights, training logs, configuration files, and evaluation metrics for over 300 models (more than 90 GB) used in this paper in the associated repository: https://dx.doi.org/10.17605/OSF.IO/36HAR

Field-specific reporting

Please select the one below that is the best fit for your research. If you are not sure, read the appropriate sections before making your selection.

☒ Life sciences ☐ Behavioural & social sciences ☐ Ecological, evolutionary & environmental sciences

For a reference copy of the document with all sections, see nature.com/documents/nr-reporting-summary-flat.pdf

Life sciences study design

All studies must disclose on these points even when the disclosure is negative.

Sample size	Sample size was dependent on the dataset, and summarized in the supplementary information. Images were labeled until >300 samples were generated or until annotators observed diminishing returns with additional labeling. Sample sizes for evaluation were determined by creating a held out test set for each dataset that consisted of 10% of the total labeled frames.
Data exclusions	No data were excluded.
Replication	Experiments involving model training were repeated a minimum of 3 times with the same configuration. Speed benchmarking experiments were repeated a minimum of 3 to 5 times depending on the range of the latency. The software, labeling and training procedures were replicated across all 5 labs represented in the paper. Attempts at replication were successful.
Randomization	Training/validation/testing dataset splits were generated randomly without replacement and kept constant across all experiments using the same dataset. When following our labeling protocol, annotators will label frames either by image features or prediction score ordering, rather than by animal identity.
Blinding	Blinding was not relevant to this study as there were not multiple experimental groups. All performance measures are computed with the same constants across architecture or experimental configurations.

Reporting for specific materials, systems and methods

We require information from authors about some types of materials, experimental systems and methods used in many studies. Here, indicate whether each material, system or method listed is relevant to your study. If you are not sure if a list item applies to your research, read the appropriate section before selecting a response.

Materials & experimental systems

n/a	Involved in the study	
☒	☐	Antibodies
☒	☐	Eukaryotic cell lines
☒	☐	Palaeontology and archaeology
☐	☒	Animals and other organisms
☒	☐	Human research participants
☒	☐	Clinical data
☒	☐	Dual use research of concern

Methods

n/a	Involved in the study	
☒	☐	ChIP-seq
☒	☐	Flow cytometry
☒	☐	MRI-based neuroimaging

Animals and other organisms

Policy information about studies involving animals; ARRIVE guidelines recommended for reporting animal research

Laboratory animals	species, strain, sex, age<br>-------------------------------------<br>Drosophila melanogaster, NM91, M+F, 3-8 days<br>Bombus impatiens, Koppert Biological Systems Common Eastern, F, 10 days<br>Mus musculus, Swiss Webster+C57BL/6J, M+F, 16 wks<br>Meriones unguiculatus, Charles River Strain Code 243, M+F, P15 to 8 months<br><br>Mice used in this study had at least 1 week of acclimation to the Princeton Neuroscience Institute vivarium in group cages with food and water ad libitum under a reversed 12/12-h dark-light cycle (light, 19:30-07:30) and habituated in the dark test room at least 30 min before experimental procedures were performed.
Wild animals	No wild animals were used in this study.
Field-collected samples	No field collected samples were used in the study.
Ethics oversight	Mice: Experimental procedures were approved by the Princeton University Institutional Animal Care and Use Committee and

Ethics oversight	conducted in accordance with the National Institutes of Health guidelines for the humane care and use of laboratory animals.<br><br>Gerbils: Experimental procedures were approved by New York University Institutional Animal Care and Use Committee and conducted in accordance with the National Institutes of Health guidelines for the humane care and use of laboratory animals.

Note that full information on the approval of the study protocol must also be provided in the manuscript.
